# Analysis of coat texture characteristics of bread wheat grains obtained from digital images

**DOI:** 10.3389/fpls.2025.1659548

**Published:** 2025-09-30

**Authors:** Dmitry R. Avzalov, Mian Abdur Rehman Arif, Evgenii G. Komyshev, Vasily S. Koval, Andreas Börner, Dmitry A. Afonnikov

**Affiliations:** ^1^ Institute of Cytology and Genetics, Siberian Branch of the Russian Academy of Sciences, Novosibirsk, Russia; ^2^ Kurchatov Genomics Center of the Institute of Cytology and Genetics of the Siberian Branch of the Russian Academy of Sciences, Novosibirsk, Russia; ^3^ Nuclear Institute for Agriculture and Biology, Faisalabad, Pakistan; ^4^ Leibniz Institute of Plant Genetics and Crop Plant Research, Seeland, Germany; ^5^ Faculty of Natural Sciences, Novosibirsk State University, Novosibirsk, Russia

**Keywords:** wheat grains, digital images, texture characteristics, genetic and environmental factors, grain aging, QTL, gene prioritization

## Abstract

**Introduction:**

The coat texture characteristics of grains in an image are informative parameters often used to classify plants into species or varieties. Intraspecific and interspecies diversity of texture parameters indicates a significant contribution of the genetic component to the formation of these traits. However, the structural and molecular properties of the grain shell, which can determine the texture in the image, have been poorly studied.

**Methods:**

Here, a comprehensive analysis of the texture characteristics of bread wheat grains from the International Triticeae Mapping Initiative (ITMI) population was performed based on their digital images.

**Results:**

The assessment of their diversity revealed two characteristic types of variability: smoothness/roughness and wrinkling along and across the grain axis. It was shown that both genotype and storage duration in the genbank contribute significantly to the formation of all grain texture characteristics investigated. Storage duration was found to be associated with an increase in grain surface roughness. A significant relationship between texture and grain germination was found for only one characteristic, GLCM (gray-level co-occurrence matrix) correlation. QTL analysis identified thirty-six additive and eight pairs of epistatic loci associated with texture traits. These loci were located on eight wheat chromosomes. Prioritization of genes in the identified loci and their functional analysis allowed us to hypothesize a possible link between grain shell texture and cell wall properties.

**Conclusion:**

The results demonstrate the genetic and environmental determinants of grain texture traits.

## 1 Introduction

The use of machine vision and digital image analysis technologies allows for the assessment of many quantitative characteristics of grain size, shape, and color (Huang et al., 2015; Zhao et al., 2022; Himmelboe et al., 2025). Grain size assessment characterizes their weight (Kim et al., 2021) and is related to plant yield (Emebiri and Hildebrand, 2023). Grain shape characteristics can serve as parameters for machine classification of plants into varieties or species (Majumdar and Jayas, 2000a; Huang and Chien, 2017; Martín-Gómez et al., 2019, Martín-Gómez et al., 2025). Color is closely related to the physiological state of grains. It characterizes the pigment composition of the shell (Del Valle et al., 2018), pathogen damage to grains (Ahmad et al., 1999), grain viability (Dell’Aquila, 2006), and grain aging processes during storage (Kibar and Kılıç, 2020; Afonnikov et al., 2022). Grain color characteristics are also used for automatic plant classification, including in conjunction with size and shape characteristics (Neuman et al., 1989; Majumdar and Jayas, 2000b; Mebatsion et al., 2013).

Quantitative assessments of grain characteristics based on digital images are used in QTL analyses or associated single nucleotide polymorphisms (SNPs) through genome wide association studies (GWAS) (Williams and Sorrells, 2014; Sakamoto et al., 2019; Alemu et al., 2020). This helps to identify genes that control grain development and its physiological properties (Jamil et al., 2025).

In addition to shape and color, the texture of objects in images can be determined, a complex characteristic that reflects the uniformity or unevenness of an object’s color pattern, as well as its regularity (Haralick et al., 1973; Galloway, 1975; Humeau-Heurtier, 2019). Grain texture characteristics are no less informative than size, shape, or color. They are often used in the classification of various plants (Majumdar and Jayas, 1999, Majumdar and Jayas, 2000c; Manickavasagan et al., 2008; Zapotoczny, 2011; Ropelewska and Jankowski, 2019; Komyshev et al., 2020; Ropelewska and Rutkowski, 2021; Gierz and Przybył, 2022; Ropelewska et al., 2022). Their use in addition to other characteristics improves the accuracy of classification (Majumdar and Jayas, 2000d). A large number of descriptors have been developed to describe the texture of objects in an image, based on the statistical properties of images, various filters, graphs, and a number of other approaches (Humeau-Heurtier, 2019). Statistical methods are most often used in the analysis of grains. These include, in particular, the gray-level co-occurrence matrix (GLCM) and the gray-level run length matrix (GLRM) (Haralick et al., 1973; Galloway, 1975). Some researchers use various components of color spaces instead of gray tones to evaluate texture characteristics (Ropelewska and Jankowski, 2019).

Despite the active study and use of grain texture characteristics in images for plant classification tasks, the structural and molecular properties of the grain shell, which can determine the texture in the image, have been poorly investigated. On the one hand, texture depends on surface morphology (smoothness, roughness, wrinkling, presence of defects). Furthermore, grain texture may be due to uneven shell coloration. There are few data on the functional role of grain surface structure, although there is evidence of its influence on seed germination in maize (Wang et al., 2025) and pea milling properties (Dijkink and Langelaan, 2002). Possible genetic mechanisms that determine grain surface structure have also been poorly studied. However, in peas, the *GRITTY* locus is known to determine seed testa roughness (Williams et al., 2024).

Previously, we proposed an approach to evaluate the characteristics of the size, shape and color of wheat grains based on the analysis of digital images obtained in the laboratory (Afonnikov et al, 2022). Characteristics for grains of 114 recombinant inbred lines (RILs) from the International Triticeae Mapping Initiative (ITMI) population harvested in 2014 were determined and search for quantitative trait loci (QTL) was performed for them (Arif et al., 2022b). Genes participating in the metabolic pathways of biosynthesis of carotenoids and flavonoids have been revealed for loci associated with shell color. Genes involved in protein ubiquitination, as well as a number of known transcription factors and enzymes involved in regulating grain development, have been identified for loci associated with grain size and shape.

Grains of plants grown in 2003, 2004, 2009, and 2014 were available in the genbank for 44 RILs. This biological material made it possible to compare the characteristics of the size, shape, and color of plant grains harvested in different years and to assess the effect of storage duration on them (Afonnikov et al, 2022). The results showed that the duration of storage correlates with changes in most of the signs of coloration, but not size/shape. The germination rates were determined for 19 lines from 2003, 2004, 2009, and 2014 harvest year seeds. Statistical analysis has shown the presence of significant correlations between germination and color characteristics characterizing the redness of the grain shell (Afonnikov et al, 2022). These results are in good agreement with known mechanisms of genetic control of grain color traits (Arif et al., 2021; Lang et al., 2024), molecular processes in seeds during aging (Gordeeva et al., 2024; Shvachko and Khlestkina, 2020), as well as known data on the relationship between redness and seed dormancy (Groos et al., 2002). The image sets we have obtained proved to be convenient for identifying the genetic and environmental determinants of wheat grain traits.

In this study, we determined 16 texture characteristics of grain coat for the same sets of grain images and performed similar analysis as in previous works (Afonnikov et al., 2022; Arif et al., 2022b). The diversity of characteristics was assessed using grains from 114 lines harvested in 2014. Analysis of grain images of 44 lines harvested in 2003, 2004, 2009 и 2014 indicated the relationship between texture characteristics and seed storage duration in the genbank. The correlation between germination and texture traits was estimated for the grains of 19 lines harvested in 2003, 2004, 2009 и 2014. 114 lines harvested in 2014 were used to identify QTLs for texture traits and possible candidate genes controlling their formation were identified. The results demonstrate the genetic and environmental determinants of grain coat texture traits.

## 2 Materials and methods

### 2.1 Seed images

Images of grains from the various accessions of the recombinant inbred lines (RILs) of the mapping population of bread wheat (*Triticum aestivum* L.) from the International Triticeae Mapping Initiative were used. The ITMI mapping population was obtained by crossing the *T. aestivum* spring wheat cultivar Opata 85 and the synthetic hexaploid spring wheat W7984 (Arif et al., 2022b). Plants of each genotype were grown in the 2003, 2004, 2009 and 2014 seasons. After harvest, the seeds were stored at IPK genbank with an -18 ± 2 °C and 8 ± 2% seed moisture content. Images were taken from previous works (Afonnikov et al., 2022; Arif et al., 2022b). The images represent grains on a white background, next to the ColorChecker calibration palette, which was used to determine the scale (x-rite ColorChecker^®^ Classic Mini, https://xritephoto.com/camera; accessed on 20 January 2022). Examples of seed images are shown in **Supplementary Figure S1** (**Supplementary File 1**).

To analyze the diversity of traits in the population and identify QTL, grain images of 114 RILs harvested in 2014 were used (Afonnikov et al., 2022; Arif et al., 2022b). Two images per RIL were obtained for this sample, each counting 15 and 5 grains, respectively. Our preliminary analysis demonstrated that this splitting does not affect the estimation of the seed traits.

Seeds from 44 lines harvested in 2003, 2004, 2009, and 2014 were used to analyze the relationship between texture traits and harvest year (Afonnikov et al., 2022). To analyze the relationship between grain germination and texture characteristics, images of seeds from 19 lines harvested in 2003, 2004, 2009, and 2014 were used (Afonnikov et al., 2022). Images for these samples included from 17 to 20 grains.

These samples were used to perform various types of analysis of grain coat texture traits as summarized in **Supplementary Figure S2** (**Supplementary File 1**): statistical relationship between traits, population diversity analysis, relationship with storage duration in genbank and germination rate, QTL identification, and gene prioritization.

### 2.2 Evaluation of seed characteristics in an image

Digital image processing was performed using the SeedCounter application (Komyshev et al., 2017), a desktop PC version supplemented with a color characteristics calculation module (Afonnikov et al., 2022). Areas corresponding to grains were identified in the image, and their size, shape, and color characteristics were evaluated as described earlier. In this study, we took 12 color characteristics, the average values of the color components of the pixels in the grain area for the RGB, HSV, Lab, and YCrCb color spaces (Afonnikov et al., 2022). Their list is given in **Supplementary Table S1** (**Supplementary File 1**).

To evaluate texture properties, we used 16 second-order characteristics determined based on gray level co-occurrence matrices (GLCM) and gray level run-length matrices (GLRM) (Haralick et al., 1973; Galloway, 1975; Majumdar and Jayas, 1999). These characteristics allow us to quantitatively describe the features of micro-relief or surface color inhomogeneities of grains, which are not always distinguishable by visual inspection. GLCM describes the spatial distribution of the brightness of neighboring pixels by evaluating the frequency of co-occurrence of certain combinations of gray-level values. GLRM evaluates sequences of pixels with the same brightness, providing information about the spatial organization of texture elements. This allows characterizing such surface properties as uniformity, contrast, and texture complexity. The list of texture features is given in **Supplementary Table S1** (**Supplementary File 1**), and the definition is given in **Supplementary Tables S2**–**S4** (**Supplementary File 1**).

When calculating texture characteristics as both GLCM (for neighboring pixels) and GLRM (for series) matrices, eight main directions are distinguished: up, down, left, right, and four diagonally. In this work, texture characteristics were determined based on grayscale images summed across all 8 directions (omnidirectional).

### 2.3 Statistical analysis of grain characteristics

A preliminary analysis of the images was conducted to exclude outliers from further consideration, i.e., grains with texture characteristics whose values deviated from the mean by more than 3 standard deviations.

To evaluate the Pearson correlation *r* of texture, size, shape, and color characteristics, grains from the 2014 harvest were analyzed (114 RILs). Based on this, the distance *d* = 1−∣*r*∣ between pairs of characteristics was calculated, and then a tree of similarity of characteristics was reconstructed using the UPGMA method. To assess the diversity of texture traits in the ITMI population for grains from the 2014 harvest, the principal component analysis (PCA) method was used based on a correlation matrix.

Grains from 44 RILs harvested in 2003, 2004, 2009 and 2014 were used to assess the contribution of genetic factors (RIL) and harvest year to the variability of texture characteristics implementing one-way analysis of variance (ANOVA). The contribution of a factor was considered significant at *p* < 0.05.

The linear correlation between the trait value and the harvest year for each grain in this sample was assessed based on the approach proposed earlier (Afonnikov et al., 2022). Data for 3460 grains were used for correlation analysis. The harvest year was coded for each grain in three ways: binary (Year01, values 0 were assigned to the years 2003 and 2004; values 1 were assigned to the years 2009 and 2014); numerical (Year, numerical values of the year were used); rank (YearRank, values 1, 2, 3, and 4 were assigned to the years 2003, 2004, 2009, and 2024, respectively). The significance of correlation between the trait and the harvest year in the three encodings was independently verified using 2000 replicates of permutation and bootstrap tests (randomization of texture trait values was used) in the sample of 3460 grains. The relationship between the trait and the harvest year was considered significant if the correlation coefficient was less than the minimum or greater than the maximum values in both randomization tests.

The statistical relationship between the trait and germination was assessed for grains obtained from 19 RILs harvested in 2003, 2004, 2009 and 2014 (1279 grains in total) using Pearson’s correlation coefficient, as was done previously (Afonnikov et al., 2022). Preliminary evaluation of the contribution of harvesting year and genotype to the germination variance demonstrated that the year (but not genotype) has significant effect. Therefore, to eliminate this effect mean germination values for the corresponding year were subtracted from the each genotype and year values. Some outlier values were removed after that in the germination data (Afonnikov et al., 2022). Before the analysis, each trait values were standardized so that the means were equal to 0 and the standard deviations were equal to 1 for all genotypes. The significance of correlation between the grain coat texture trait and the germination rate was assessed by randomization tests as described above.

Statistical data processing was performed using Python 3.10 software (SciPy, sklearn, pandas libraries).

### 2.4 QTL analysis of grain texture characteristics

Grains from 114 RILs harvested in 2014 were used for QTL analysis. Mean values of the grain texture characteristics for each RIL served as input data.

Experimental procedures to obtain SNPs in ITMI plants for QTL analysis were described in (Arif et al., 2021).

To capture the variance explained by the molecular markers such as SNPs mapped to any genome, a refined method known as “inclusive composite interval mapping” was used as implemented in the QTLIciMapping 4.2.53 (http://www.isbreeding.net/(latest released in September 2019). This method currently considered as the most modern method of QTL detection (Arif et al., 2021). It was used successfully to detect several QTLs for Fusarium head blight (Sgarbi et al., 2021) and seed longevity (Arif et al., 2022a) in wheat and germination related traits in tobacco (Agacka-Mołdoch et al., 2021) applying the QTLIciMapping tool. Therefore, we convened the IciMapping 4.2.53 to detect the putative additive QTLs of the traits under consideration applying the inclusive composite interval mapping (ICIM) command where 1.0 cM was the walking speed. An LOD score of > 2.0 ≤ 3 was applied to detect QTLs as significant and > 3.0 as highly significant (Meng et al., 2015).

In order to discover digenic epistasis QTLs to find clues for latent variation, the ICIM-EPI command was used where LOD was kept 5.0 cM. Here, the epistasis QTLs with LOD ≥5 and explaining ≥ 5% phenotypic variance were reported. All QTLs were assigned names according the rules set out in the Catalog of Gene Symbols (McIntosh et al., 2008). Epistasis QTLs were visualized using “circlize” package in R (Gu et al., 2014).

In addition to 16 texture traits for QTL identification, we used their two linear combinations represented by PC1 and PC2 from the PCA analysis for seeds harvested in 2014. (see above). The QTL locations for texture traits were compared with QTLs for traits such as size, shape, and color of grains in images from our previous work (Arif et al., 2022b).

### 2.5 Gene prioritization

Gene identification in QTL regions, their functional annotation, and prioritization were performed according to previously described procedures (Arif et al., 2022b). The sequences of markers delimiting QTLs were aligned on IWGS 2.1 wheat genome sequence (Zhu et al., 2021). Genome sequence and annotation data were obtained from URGI (https://urgi.versailles.inra.fr/download/iwgsc/IWGSC_RefSeq_Assemblies/v2.1; accessed on 10 January, 2022). Only ‘high confidence’ gene annotations were considered. Marker sequences were obtained from reference (Wang et al., 2014) and Gramene marker Database (https://archive.gramene.org/markers/; accessed on 10 January, 2022) (Tello-Ruiz et al., 2021). Marker sequences were aligned using blastn of the BLAST+ package (Camacho et al., 2009) using e-value=1e-17 (other parameters were set by default). Marker locations were selected by choosing appropriate chromosome and highest sequence identity with the reference. Search for genes and their functional analysis were performed only for QTLs which have both left and right markers mapped on the reference genome.

Genes located within marker borders were selected by expression level in the grain (TPM>=1). For this purpose, wheat gene expression data from the expVIP database (Borrill et al., 2016) were used. Data in text format were downloaded from URGI (https://urgi.versailles.inra.fr/download/iwgsc/IWGSC_RefSeq_Annotations/v1.1/iwgsc_refseqv1.1_rnaseq_mapping_2017July20.zip; accessed on 10 January, 2022). We used data from RNA-seq experiments in which the column ‘High level tissue’ contains ‘grain’ term. Additional conversion was performed between annotation ver. 2.1 (genome) and 1.2 (transcriptome) gene IDs.

Since there was no prior knowledge about possible molecular mechanisms related to seed texture characteristics in wheat, two approaches for functional annotation and gene prioritization were used. First, full list of QTL related genes expressed in seeds was analyzed by DAVID web service, https://davidbioinformatics.nih.gov/, accessed on 12 April, 2025 (Sherman et al., 2022). Functional clusters and functional charts of genes were obtained. Clusters and functional categories were selected using *p*-values corrected for multiple hypothesis testing (Benjamini correction and FDR < 0.05). Second, sequences of selected genes were used to search for KEGG Orthology (KO) annotation by BlastKOALA and GhostKOALA web-services (Kanehisa et al., 2016). List of KO IDs was compared with orthologous groups of genes related to seed development in Arabidopsis and rice according to literature data, see details in (Arif et al., 2022b).

## 3 Results

### 3.1 Correlations between texture characteristics and other seed traits

**Figure 1:**
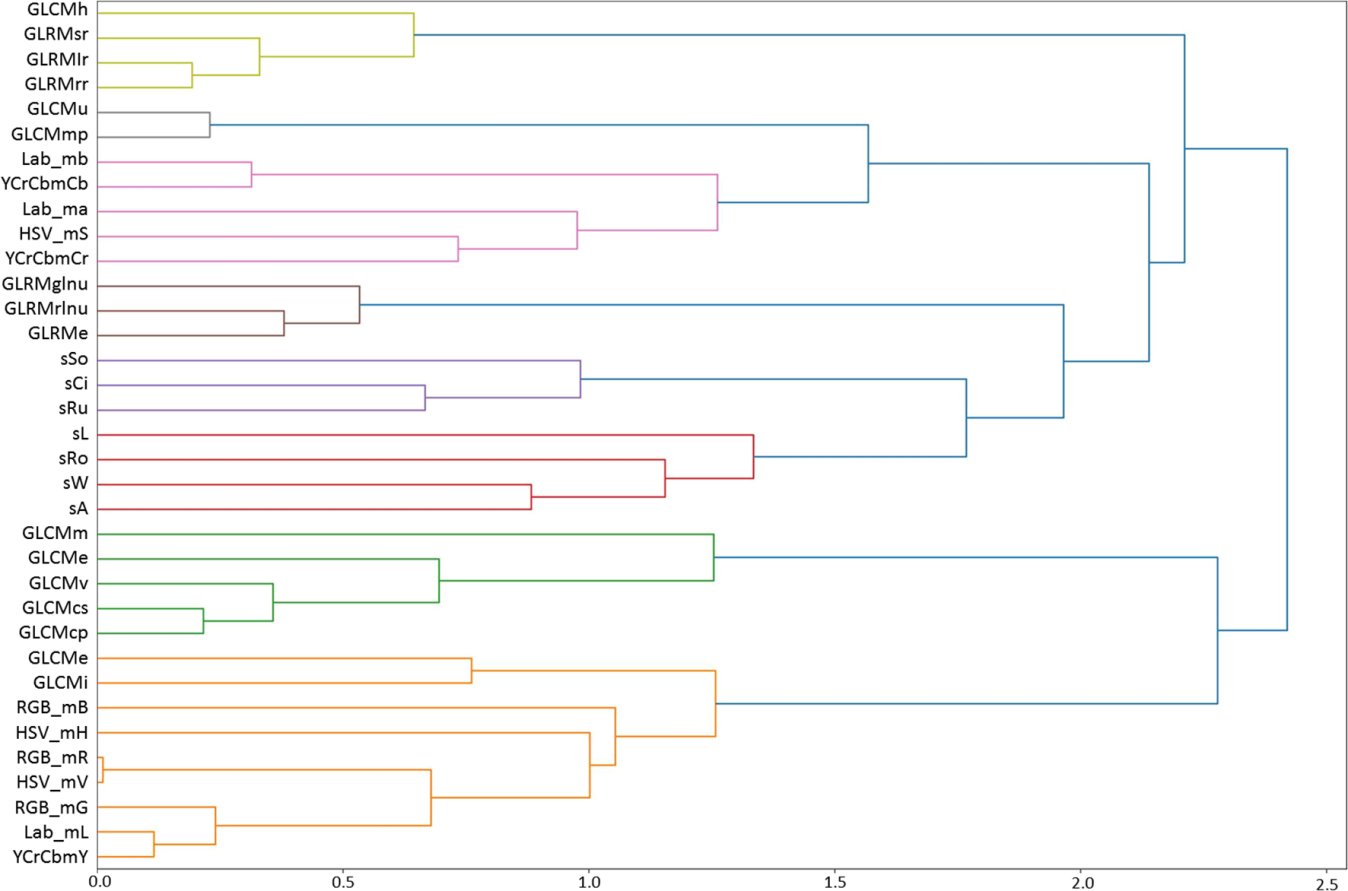
Hierarchical clustering of grain traits, including texture traits, based on Pearson’s correlation coefficient estimated from their variability in 114 wheat lines of the ITMI population. The proximity scale is shown on the X-axis. Trait clusters in the diagram are highlighted in different colors.

The values of Pearson’s correlation coefficients for pairs of traits are given in **Supplementary Table S5** (**Supplementary File 1**). The table shows that significant correlation coefficients are more often observed for pairs within groups of color, size/shape, and texture traits, with several texture traits having significant correlation coefficients with color traits. The tree of grain traits similarity based on these coefficients is shown in **Figure 1**. Eight clusters are distinguished in the tree (at a clustering threshold of *d* = 1.5). The first cluster (top to bottom, light green) includes four grain texture features, three of which are based on GLRM (GLRMsr, GLRMlr, GLRMrr), and one is calculated based on the GLCM matrix (GLCMh). The second cluster (gray) includes two texture features (GLCMu, GLCMmp). The third cluster (pink) includes five grain color features that characterize color (Lab_mb, YCrCb_mCb, Lab_ma, HSV_S, YCrCb_Cr). The next cluster (brown) includes texture features based on GLRM (GLRMglnu, GLRMrlnu, GLRMe). The next two clusters, lilac and red, include shape and size features, respectively. The green cluster includes five texture features based on GLCM calculation. Finally, the orange cluster includes nine features. Of these, two outlying features characterize texture (GLCMc, GLCMi). The remaining features form a tight cluster and include three components of the RGB space; the remaining ones, except for HSV_mH, characterize the lightness/brightness of pixels.

Thus, the clusters on the dendrogram correspond to several interrelated groups of characteristics that describe the shape, size, color, lightness, and texture of the grains. The assignment of characteristics to clusters reflects their common biological nature. Note that texture characteristics are grouped into several clusters. The exceptions are GLCMc and GLCMi, which fall into the brightness trait cluster but are simultaneously quite distant from it. This means that grain texture traits reflect specific surface characteristics that are not related to shape or size and are to some extent related to the lightness of the grain shell (**Figure 1**).

### 3.2 Diversity of ITMI population based on texture characteristics

**Figure 2:**
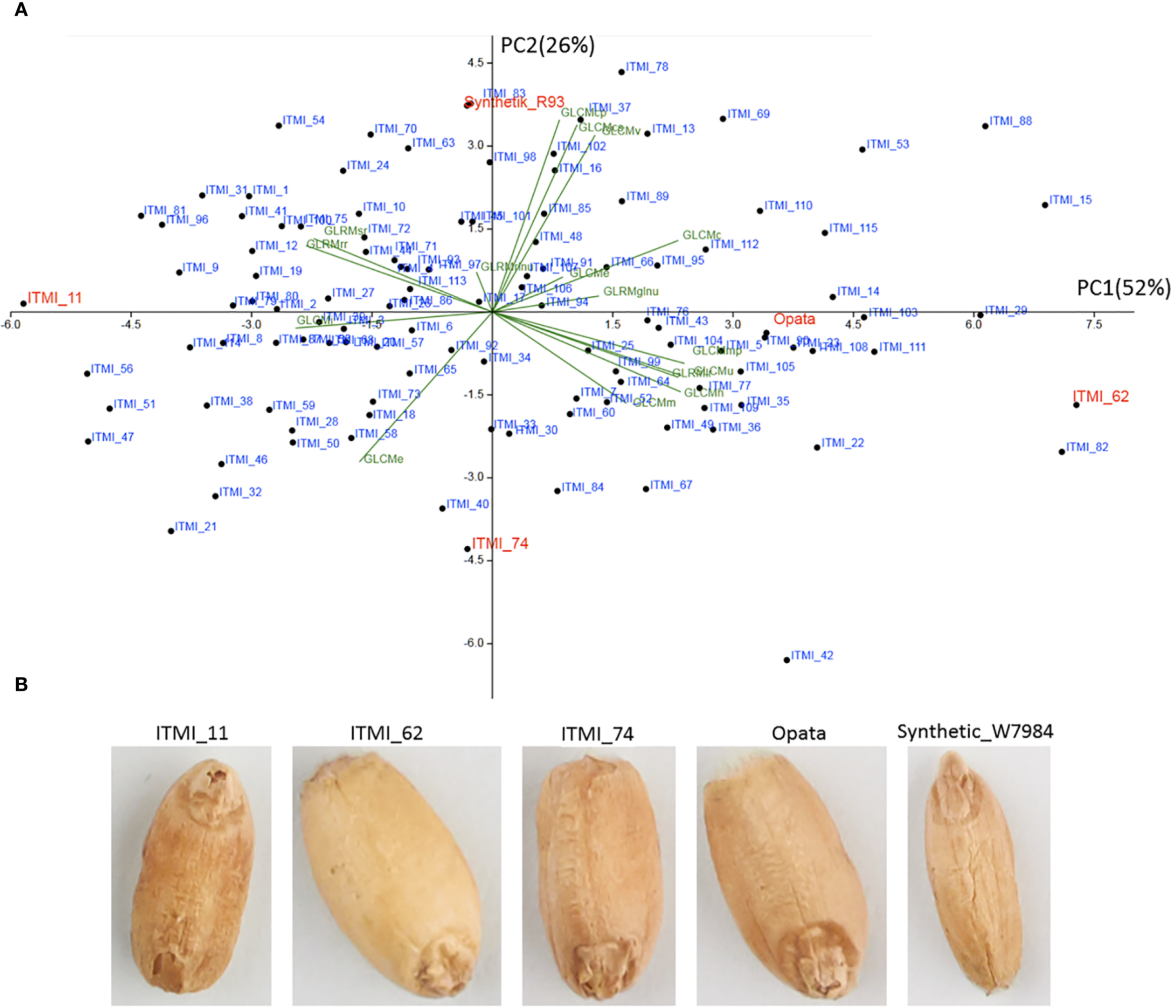
**(A)** PCA diagram in the space of GLCM and GLRM texture features. The PC1 and PC2 axes correspond to the first and second principal components, with the corresponding dispersion shares indicated in parentheses. The points correspond to wheat lines, with texture feature projections shown in green. Lines with extreme component values, including parental genotypes, are shown in red. **(B)** Grain images for lines with extreme component values.

We analyzed the diversity of grains in the ITMI population based on texture characteristics using PCA. The results are shown in a scatter plot for the two principal components (**Figure 2**). These two components account for 78% of the total variance. The diagram demonstrates the wide variability of the ITMI population in terms of texture, with no clusters standing out.

The first principal component (PC1) explains half of the total variance and shows a high positive correlation with features such as GLRMglnu, GLCMc, and GLRMe. This component also shows a high negative correlation with the GLCMi feature (**Figure 2**).

It should be noted that high values of this component are observed for the ITMI_62 line (**Figure 2A**), whose grains appear to be the smoothest (**Figure 2B**). Opata grains also have positive values for this component, and their surface also appears smooth with small wrinkles. Conversely, for the ITMI_11 line, the value of this component is the lowest and negative (**Figure 2A**). In the image, these grains appear to be the roughest (**Figure 2B**). It can be assumed that the first component reflects the smoothness of the grain: the higher its value, the smoother the grain surface; the lower the value, the rougher the grain surface. Note that smooth grains appear lighter in color than rough ones (ITMI_62, ITMI_11, **Figure 2B**), which may partly explain the high correlation coefficients between some texture and lightness features in **Supplementary Table S5** (**Supplementary File 2**) and **Figure 1**.

**Figure 3:**
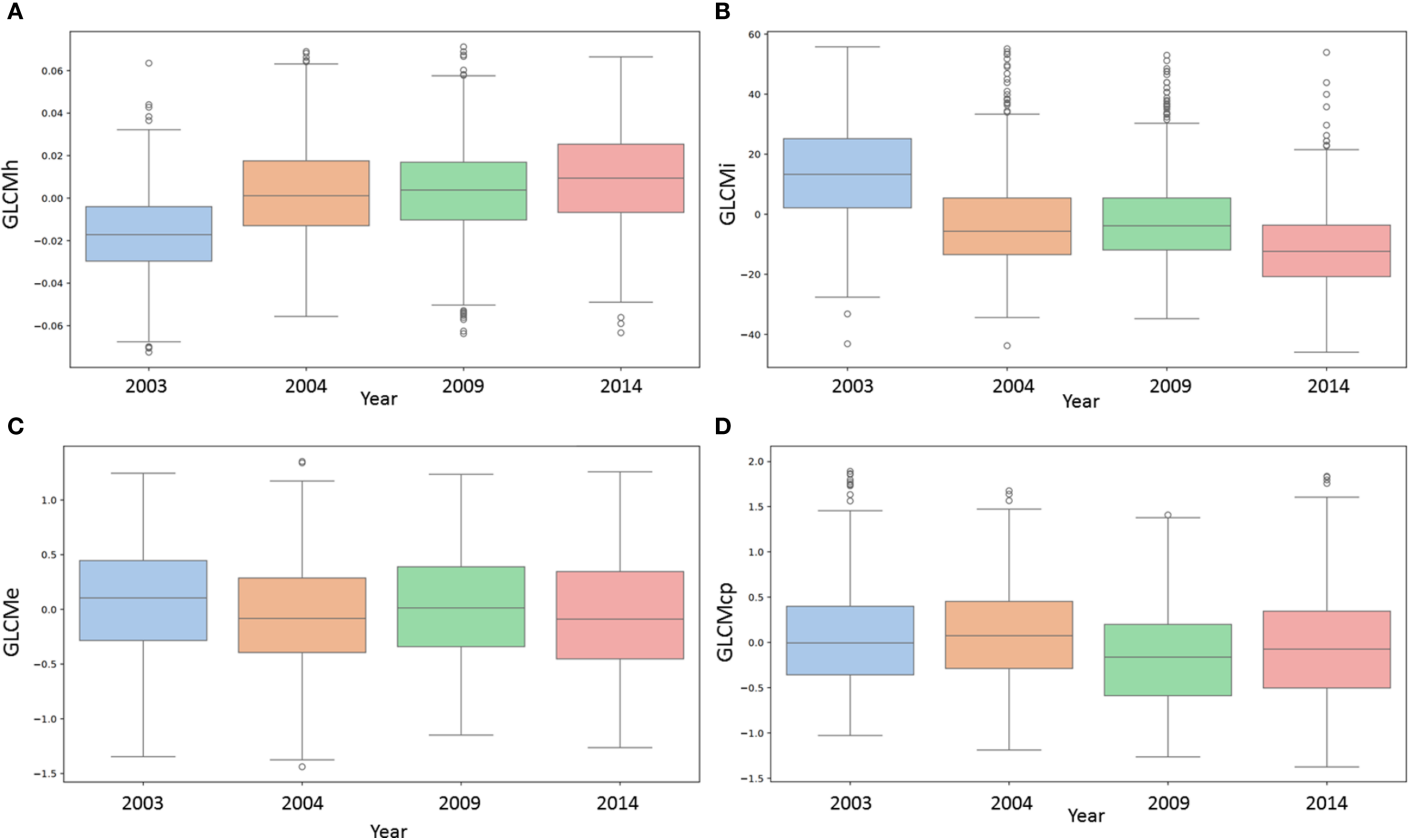
Bar plots showing the dependence of four texture characteristics of wheat grains on the year of harvest. **(A)** GLCMh (GLCM homogeneity); **(B)** GLCMi (GLCM inertia); **(C)** GLCMe (GLCM entropy); **(D)** GLCMcp (GLCM cluster prominence). The horizontal axis shows the years of storage (2003, 2004, 2009, 2014), and the vertical axis shows the values of the characteristics.

The second principal component (PC2) explains a quarter of the total variance and shows a high positive correlation with features such as GLCMcp, GLCMcs, GLCMv, and GLRMrlnu. A negative correlation with this component is observed for the GLCMe feature. Interestingly, a high positive value for this component is observed for the Synthetic_W7984 sample, whose grains appear wrinkled, with wrinkles extending along the grain (**Figures 2A, B**). Low values for this component are characteristic of the ITMI_74 line (**Figures 2A**). Small transverse folds are observed for its grains (**Figure 3B**). It can be assumed that the second component reflects the folding of the grain surface. At the same time, its values probably characterize the direction of the grain surface wrinkles: low values correspond to wrinkles directed across the grain, and high values correspond to wrinkles directed along the grain.

**Figure 2** demonstrates the high diversity of grain texture in the ITMI population and allows to distinguish two main types of variability: smoothness/roughness and wrinkling along and across.

### 3.3 Contributions of genetic components and harvest year to the diversity of textural traits

Data for various harvest years were presented for 44 lines. ANOVA tests were used to estimate the contributions from both genetic and harvest year factors to the variability of traits related to grain texture in the image. The results are presented in **Supplementary Table S6** (**Supplementary File 1**). Results demonstrated that the variability of all 16 traits is due to a significant contribution of both genotype and harvest year. The *p*-values were significantly less than 5% for all 16 traits and both factors. The highest *p*-values were observed for the “genotype” factor and the GLRMglnu and GLRMe traits (0.00047 and 0.00043, respectively). This ensures the reliability of the contribution of the two factors, even when taking into account the correction for multiple comparisons.

Thus, the results of the analysis confirm that the diversity of grain texture traits in the studied wheat samples is influenced by both genetic and environmental (harvest year) factors.

### 3.4 The relationship between the harvest year and texture characteristics

For 44 ITMI lines, trends in grain texture variability depending on the harvest year were assessed based on Pearson’s correlation coefficient with three variants of numerical representation of the harvest year: binary Year01, rank YearRank, and numerical Year. For each representation of the harvest year, we conducted two randomization tests with 2,000 replicates, permutation and bootstrap. With their help, we determined the minimum and maximum confidence limits of the correlation coefficient.

Results are shown in **Table 1**. Only two of the 16 texture characteristics do not have a significant correlation between their values and the harvest year. The correlation coefficients for the GLCMv and GLCMe features are less than 0.07 in magnitude. In addition, for the GLCMcs feature, the correlation coefficient with the YearRank parameter (0.07) only slightly exceeds the threshold value (0.06) in magnitude.

**Table 1:** Evaluation of Pearson correlation coefficients between grain texture characteristics and harvest year, presented in three encodings.

Trait	Year01	Year	YearRank	PermYearRank		BootstrapYearRank	
				Min	Max	Min	Max
GLCMcp	-0.16	-0.12	**-0.11**	-0.05	0.07	-0.05	0.07
GLCMcs	-0.11	-0.07	**-0.07**	-0.06	0.06	-0.05	0.06
GLCMc	0.20	0.26	**0.30**	-0.06	0.05	-0.06	0.05
GLCMe	-0.01	-0.04	-0.06	-0.06	0.06	-0.06	0.06
GLCMh	0.27	0.29	**0.33**	-0.06	0.06	-0.07	0.06
GLCMi	-0.33	-0.39	**-0.44**	-0.06	0.06	-0.07	0.06
GLCMmp	0.06	0.07	**0.12**	-0.06	0.07	-0.07	0.06
GLCMm	0.11	0.09	**0.11**	-0.05	0.07	-0.05	0.06
GLCMuy	0.15	0.14	**0.17**	-0.07	0.06	-0.04	0.06
GLCMv	-0.06	-0.03	-0.02	-0.05	0.06	-0.06	0.05
GLRMe	0.14	0.11	**0.12**	-0.07	0.06	-0.06	0.06
GLRMglnu	0.15	0.12	**0.13**	-0.06	0.08	-0.07	0.06
GLRMlr	0.21	0.24	**0.28**	-0.06	0.07	-0.06	0.06
GLRMrlnu	0.13	0.10	**0.10**	-0.06	0.06	-0.05	0.06
GLRMrr	-0.22	-0.25	**-0.28**	-0.06	0.06	-0.06	0.07
GLRMsr	-0.20	-0.22	**-0.26**	-0.07	0.06	-0.06	0.06

The four right-hand columns show the minimum and maximum threshold values of the correlation coefficients obtained based on permutation (PermYearRank) and bootstrap (BootstrapYearRank) tests for the rank coding of the harvest year. Values in bold for YearRank parameter are significant according to randomization tests.

In other cases, the correlation coefficient of the trait with the YearRank variable is equal to or greater than 0.1. The highest positive correlation coefficients (greater than 0.28) are observed for traits such as GLCMc, GLCMh, and GLRMlr. These results indicates that these traits are greater for grains from a later harvest year (or shorter storage period in the gene bank). Interestingly, these three traits on the principal component diagram (**Figure 2**) are collinear with the first principal component, which can be interpreted as an increase in grain smoothness (**Table 1**).

The lowest correlation coefficients (< -0.26) are demonstrated by GLRMsr, GLRMrr, and GLCMi. The latter has the highest correlation coefficient among all of them with the YearRank feature (-0.44). Negative correlation coefficients mean that the lower the harvest year (and therefore the longer the storage time of the grains), the greater the value of the feature. Note that the three features indicated in the principal component diagram (**Figure 2**) are opposite in relation to the PC1 component (associated with grain smoothness). Thus, an increase in the value of these features can be interpreted as an increase in grain roughness.

**Table 1** shows that the correlation coefficients of the features with the harvest year, determined by different year encodings, are generally consistent with each other. For example, for GLCMh, the correlation coefficient with Year01 was *r* = 0.269, with Year *r* = 0.292, and with YearRank *r* = 0.333. At the same time, the YearRank coding generally shows the lowest absolute values of correlations with features. Therefore, selecting the threshold for randomization tests based on it gives more conservative estimates of significance.

Examples of the relationship between the magnitude of certain traits and the harvest year are shown as box plots in **Figure 3** (for other traits, they are shown in **Supplementary Figure S3**, **Supplementary File 1**). These graphs clearly show trends in trait variability depending on the year if the correlation coefficient estimate differs significantly from 0. For example, for the GLCMh trait (**Figure 3A**), there is a steady increase depending on the harvest year. This is consistent with the high correlation coefficient values (**Table 1**).

For the GLCMi trait, on the contrary, the opposite trend is observed: as the harvest year increases, the value of the trait decreases (**Figure 3B**). This is consistent with the negative value of the GLCMi correlation coefficients with the harvest year (**Table 1**). It should be noted that higher GLCMi values correspond to greater grain roughness (**Figures 2A, B**) and are higher for earlier harvest years, i.e., for longer grain storage periods (**Figure 3**).

For the GLCMe trait (**Figure 3C**), the values for different harvest years differ, but no trend with increasing harvest year is observed. This is consistent with the results in **Table 1**: there is no significant statistical relationship between this trait and the harvest year. In **Figure 3D**, the values of the GLCMcp trait for the harvest years 2003 and 2004 are slightly higher than the values for 2009 and 2014. There is a noticeable downward trend with increasing harvest year, but it is less pronounced than for the GLCMh and GLCMi traits. This is also consistent with the data in **Table 1**: the absolute value of the correlation coefficient with the harvest year for GLCMcp is less than for GLCMh and GLCMi.

Summarizing the results presented, it can be assumed that the correlations between texture characteristics and harvest year that we have identified reflect, in general, an increase in the roughness of wheat grain coat as the storage period in the genbank increases. Thus, the results show that storing grains in a genbank leads to changes in their texture. These changes may reflect structural or metabolic changes in the grain shell.

### 3.5 The relationship between grain germination and their textural characteristics

We assessed the relationship between grain texture characteristics and germination rates. The results are presented in **Supplementary Table S7** (**Supplementary File 1**). They show that only one characteristic, GLCMc (GLCM correlation), meets the criteria for a significant deviation from 0. At the same time, its correlation coefficient with germination (0.098) only slightly exceeds the threshold obtained for the permutation (0.087) and bootstrap (0.097) tests. We note another feature, GLCMi (GLCM inertia). For it, the correlation coefficient with similarity was -0.102, which is slightly less than the lower threshold for the bootstrap test (-0.095), but exceeds the lower threshold for the permutation test (-0.104). Thus, this feature satisfies the criterion of a significant deviation from 0 based on the results of only one randomization test. Interestingly, both GLCMc and GLCMi correlate with grain lightness features (see **Figure 1**). At the same time, the GLCMi was interpreted as characteristics of grain shell roughness (**Figure 2**).

For the remaining texture parameters, the correlation coefficients between the normalized values of the trait and germination are within the ranges obtained from randomization tests. Thus, it can be concluded that the statistical relationship between grain texture traits and their germination is weak.

### 3.6 QTL mapping for texture traits

**Figure 4:**
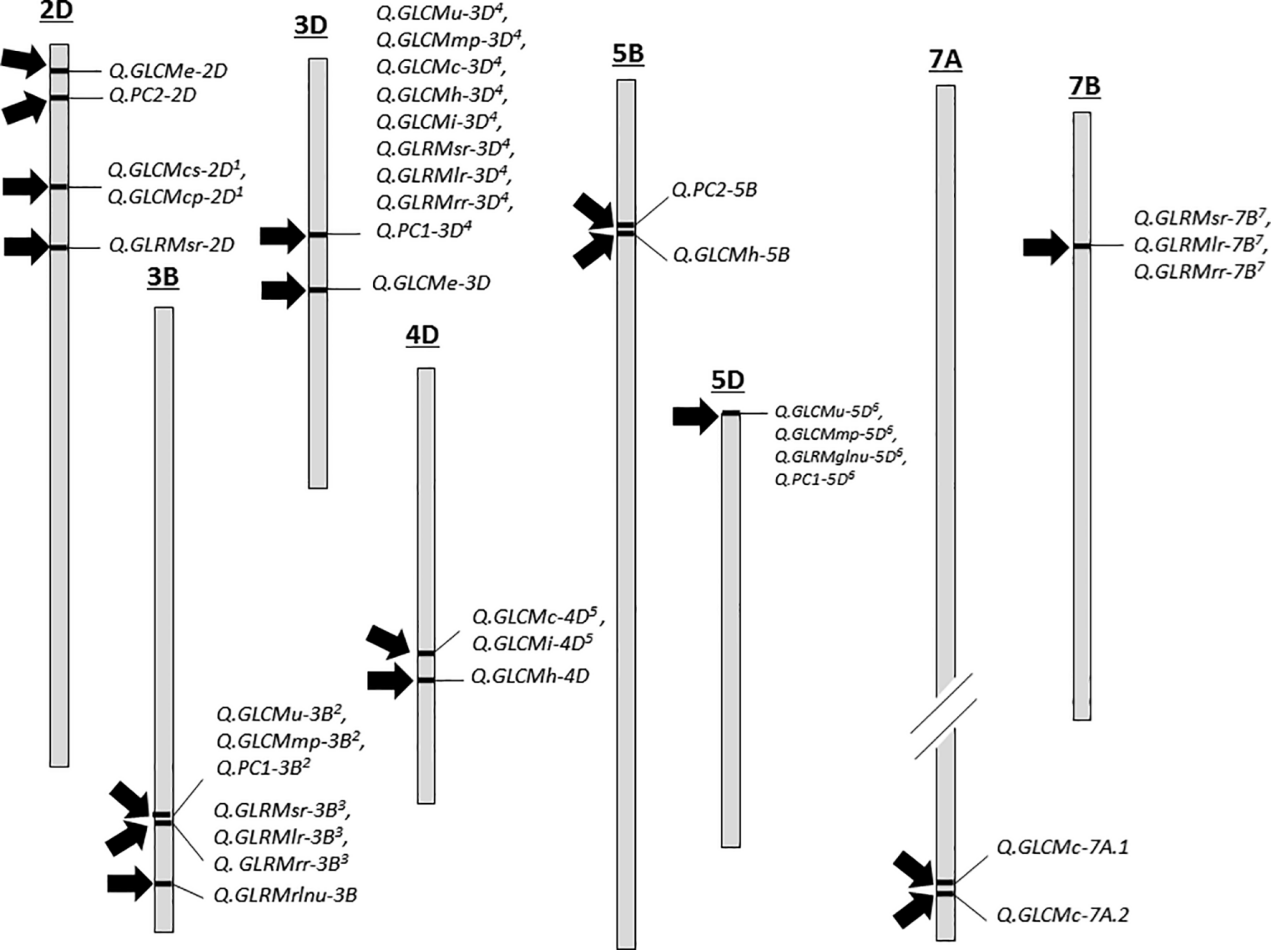
QTL distribution of various grain texture traits. Each chromosome is drawn to an approximate where each 1 cm distance = ~ 80 cM and chromosome 7A is shown smaller in comparison to its actual length as indicated by small cut. QTL with similar superscripts are identical loci. For details, see **Supplementary Table S8** (**Supplementary File 2**).

For seed texture traits, a total of 36 texture related QTLs were discovered on chromosomes 2D, 3B, 3D, 4D, 5B, 5D, 7A and 7B where majority were overlapped with other QTLs (**Supplementary Table S8**, **Supplementary File 2**; **Figure 4**). For example, there were four QTLs (*Q.GLCMe-2D*, *Q.GLCMcs-2D^1^*, *Q.GLCMcp-2D^1^* and *Q.GLRMsr-2D*) on chromosome 2D. These QTLs were related to GLCMe, GLCMcs, GLCMcp and GLRMSr where the log of odds (LOD) values ranged from 2.5 to 6.1 and the phenotypic variation explained (PVE) varied from 6.19 to 17.52%. In addition, the QTLs for GCLMCs and GCLMcp overlapped. Likewise, on chromosome 3B, there were six QTLs (*Q.GLCMu-3B^2^*, *Q.GLCMmp-3B^2^*, *Q.GLRMsr-3B^3^*, *Q.GLRMlr-3B^3^*, *Q.GLRMrr-3B^3^* and *Q.GLRMrlnu-3B*) linked with GCLMu, GCLMmp, GLRMsr, GLRMlr, GLRMrr and GLRMrlnu. Here the LOD ranged from 2.5 to 3.30 and the PVE ranged from 8.58 to 11.87% whereas all the QTLs except *Q.GLRMrlnu-3B* were nearly overlapping with each other (**Figure 4**).

On chromosome 3D there were nine QTLs (related to *Q.GLCMu-3D^4^*, *Q.GLCMmp-3D^4^*, *Q.GLCMc-3D^4^*, *Q.GLCMh-3D^4^*, *Q.GLCMi-3D^4^*, *Q.GLRMsr-3D^4^*, *Q.GLRMlr-3D^4^*, Q*.GLRMrr-3D^4^* and *Q.GLCMe-3D*) GLCMu, GLCMmp, GLCMc, GLCMh, GLCMi, GLRMsr, GLRMlr, GLRMrr, and GLCMe and all of them except GLCMe were at the same location. The PVE by these QTLs ranged from 8.5 to 33.23% and the maximum LOD was 9.56. On chromosome 4D, there were three QTLs (*Q.GLCMc-4D^5^*, *Q.GLCMi-4D^5^* and *Q.GLCMh-4D*) related to GLCMc, GLCMi and GLCMh that explained 4.48 to 10.24% variation and the LOD remained between 2.67 and 3.41. On chromosome 5B there was one single QTL *Q.GLCMh-5B*) related to GLCMh responsible for 9.31% variation with an LOD value of 3.40. On chromosome 5D, there were three QTLs (*Q.GLCMu-5D^6^*, *Q.GLCMmp-5D^6^* and *Q.GLRMglnu-5D^6^*) at the exact location related to GLCMu, GLCMmp and GLRMglnu responsible for > 10% variation and their LOD ranged from 2.53 to 3.52. There were two separate QTLs (*Q.GLCMc-7A.1* and *Q.GLCMc-7A.2*) related to GLCMc on chromosome 7A with LOD values of 9.89 and 14.36 explaining 15.14 and 24.38% variation. Finally, on chromosome 7B, we detected three overlapping QTLs (*Q.GLRMsr-7B^7^*, *Q.GLRMlr-7B^7^* and *Q.GLRMrr-7B^7^*) for GLRMsr, GLRMlr and GLRMrr where the LOD value was >3 and the PVE ranged from 8.76 to 10.84.

To capture additional variation, we also used the first two principal components as trait values and performed the QTL mapping. Interestingly, we detected five QTLs (three with PCI and two with PC2) (**Supplementary Table S8**, **Supplementary File 2**). The QTLs of PC1 on chromosomes 3B, 3D and 5D (*Q.PC1-3B^2^*, *Q.PC1-3D^4^* and *Q.PC1-5D^6^*) overlapped exactly with the texture related QTLs. On the other hand, the two QTLs with PC2 (*Q.PC2-2D* and *Q.PC2-5B*) on chromosomes 2D and 5B did not overlap with other QTLs. These QTLs explained > 17% phenotypic variance and their LOD values were also > 5.

**Figure 5:**
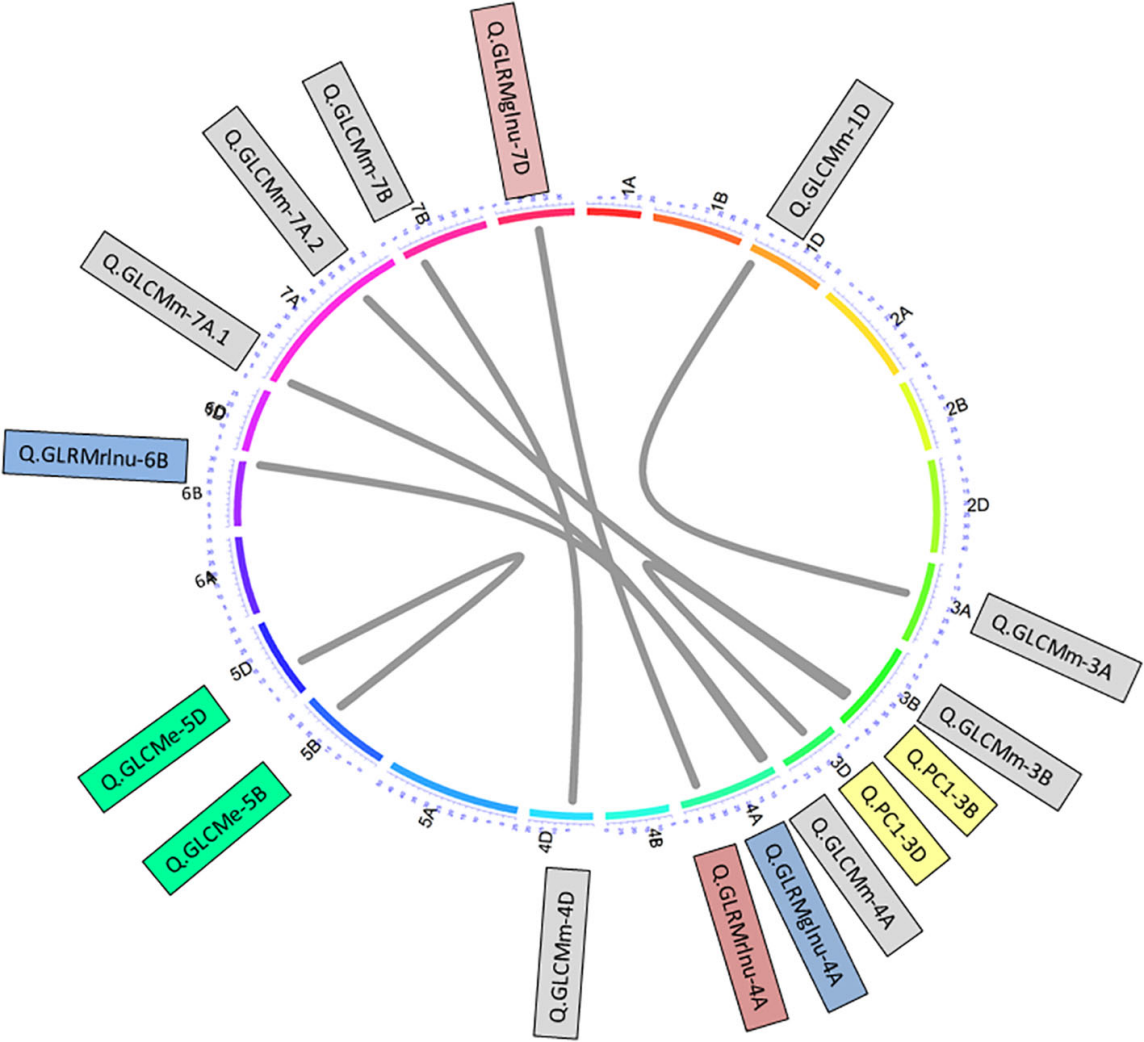
Epistasis QTL network of grain texture traits. Outer circular plot represents the hexaploid genome arranged in chromosomes (chrs) 1–21 (1A–7D) in clockwise direction. Numbers on colored outer circle represents cM on respective chrs. Grey-colored connections represent epistasis QTL controlling different traits. Similar shaded QTL indicated QTL of similar traits.

Additionally, epistatic analyses further detected a total of eight pairs that further explained up to 52.66% variation individually (**Supplementary Table S9**, **Supplementary File 2**; **Figure 5**). From trait perspective, there were four epistatic pairs of QTLs detected for GLCMm on chromosomes 1D-3A, 3B-7A, 4A-7A and 4D-7B. The variation explained by these pairs ranged from 9.79 to 21.96%. One pair for GLCMe was detected on chromosomes 5B-5D responsible for 17.95% variation. Another pair was detected for GLRMglnu on chromosomes 4A-7D causing 21.92% variation in trait expression. Further, another pair was detected for GLrMrlnu on chromosomes 4A-6B. This pair was responsible for 20.58% variation in the trait. We also detected an epistatic QTL pair for PC1 on chromosomes 3B-3D that explained > 50% variation in PC1 (**Figure 5**).

The results of comparing the positions of QTL for texture traits on chromosomes with QTL for grain size/shape and color traits in images are shown in **Supplementary Table S10** (**Supplementary File 2**). This table shows that QTL for texture traits do not coincide with any of the QTL for grain size and shape traits in the wheat genome. However, there is an overlap with a large number of grain color traits in two locations. The first region is located on chromosome 3B at positions 299.179–300.179 cM (delimited by markers Excalibur_c5309_286 and BS00085434_51).

In this location, three QTLs for texture characteristics (*Q.GLRMsr-3B^3^*, *Q.GLRMlr-3B^3^*, *Q.GLRMrr-3B^3^*) and 14 QTLs for color traits were found. Second region is located on 3D chromosome at 100–102 cM (delimited by SNPs CAP12_c2615_128 and BS00067163_51). In this location, 9 QTLs for texture characteristics (*Q.GLCMu-3D^4^*, *Q.GLCMmp-3D^4^*, *Q.GLCMc-3D^4^*, *Q.GLCMh-3D^4^*, *Q.GLCMi-3D^4^*, *Q.GLRMsr-3D^4^*, *Q.GLRMlr-3D^4^*, *Q.GLRMrr-3D^4^* and *Q.PC1-3D^4^*) and 34 QTLs for color traits were observed.

Other notable QTLs for texture characteristics (*Q.GLCMu-3B^2^*, *Q.GLCMmp-3B^2^* and *Q.PC1-3B^2^*) were located on 3B chromosome at 297.179 cM (delimited by SNPs Excalibur_c36725_96 and Ku_c24974_674). This region is close to seven QTLs for the color traits (position 298.179 cM, SNPs Ku_c24974_674 and Excalibur_c5309_286) and has common SNPs Ku_c24974_674 for these loci.

The remaining QTLs for texture traits do not share common locations on chromosomes with QTLs for color traits.

### 3.7 Functional analysis and prioritization of genes within QTL related to texture traits

To prioritize genes based on highly significant QTLs (LOD>3), we selected 27 QTLs out of 36. Of these, 23 QTLs were related to texture traits, two to their linear combination PC1 (on chromosomes 3B and 3D), and two to PC2 (on chromosomes 2D and 5B). In the wheat genome, these QTLs correspond to 13 regions. For nine of them, we determined the coordinates of the left and right markers in the IWGS 2.1 genome sequence. The sizes of the regions ranged from 2 to 80 Mb. Using genome annotation, 1,688 wheat genes were identified that are localized within the boundaries of these regions. The number of genes per region ranged from 15 (QTLs on chromosome 3B at position 297.179) to 526 (15 QTLs on chromosome 4D at position 164.488). Lists of selected QTLs, their position in the genome sequence, and lists of corresponding genes are provided in **Supplementary Table S11** (**Supplementary File 2**). Of the 1,688 wheat genes, 1,213 genes were selected based on their expression level in grain. Their list is provided in **Supplementary Table S12** (**Supplementary File 2**).

These genes were analyzed for functional enrichment using the DAVID service. **Table 2** presents information on two functional clusters of genes identified by us that demonstrate a statistically significant association with functional annotation terms. The first cluster includes genes whose function is associated with the INTERPRO domains IPR000490 (Glycoside hydrolase family 17; 13 genes) and IPR044965 ‘Glycoside hydrolase family 17, plant’ (detected for 12 genes), as well as the term GO:0004553 ‘hydrolase activity, hydrolyzing O-glycosyl compounds’ (detected for 21 genes).

**Table 2:** Results of analysis by DAVID web-service for the gene Annotation Clusters 1 and 2 (Enrichment Score 4.29 and 3.29, respectively).

Category	Term ID	Term	Number of genes	%	P-value	Benjamini	FDR
Cluster #1							
INTERPRO	IPR000490	Glycoside hydrolase family 17	13	1.2	2E-06	**2E-04**	**2E-04**
INTERPRO	IPR044965	Glycoside hydrolase family 17, plant	12	1.1	5E-07	**5E-04**	**5E-04**
GOTERM_MF_DIRECT	GO:0004553	Hydrolase activity, hydrolyzing O-glycosyl compounds	21	1.9	5E-05	**0.03**	**0.03**
INTERPRO	IPR017853	Glycoside hydrolase superfamily	22	2.0	5E-04	0.16	0.16
GOTERM_BP_DIRECT	GO:0005975	Carbohydrate metabolic process	28	2.6	2E-03	0.50	0.50
UP_KW_MOLECULAR_FUNCTION	KW-0326	Glycosidase	23	2.1	0.01	0.29	0.29
Cluster #2							
GOTERM_BP_DIRECT	GO:0046513	Ceramide biosynthetic process	7	0.7	5E-06	**3E-03**	**3E-03**
INTERPRO	IPR016439	Lag1/Lac1-like	5	0.5	2E-05	**1E-02**	**1E-02**
GOTERM_MF_DIRECT	GO:0050291	Sphingosine N-acyltransferase activity	5	0.5	3E-05	**1E-02**	**1E-02**
UP_SEQ_FEATURE	DOMAIN	TLC	5	0.5	9E-04	0.16	0.16
INTERPRO	IPR006634	TLC-dom	5	0.5	1E-03	0.37	0.37
SMART	SM00724	TLC	5	0.5	1E-03	0.24	0.24
UP_KW_CELLULAR_COMPONENT	KW-0256	Endoplasmic reticulum	17	1.6	0.01	0.24	0.24
GOTERM_CC_DIRECT	GO:0005789	Endoplasmic reticulum membrane	15	1.6	0.28	1	1

Significant *p*-values after Benjamini correction and FDR shown in bold.

The second cluster contains three significant terms. All of them are related to ceramide biosynthesis: ‘ceramide biosynthetic process’ (GO:0046513, 7 genes), ‘Lag1/Lac1-like’ domains (IPR016439, 5 genes), sphingosine N-acyltransferase activity (GO:0050291, 5 genes).

**Supplementary Table S13** (**Supplementary File 2**) presents annotation terms that are significantly represented in the sample of genes localized in regions of highly significant QTLs according to DAVID data. In addition to the terms presented in **Table 2**, the table contains Uniprot annotation terms ‘Disordered region’ (detected for 635 genes). Another group of genes is characterized by the annotation of UP_SEQ_FEATURE sequences (COMPBIAS, Basic residues).

Thus, the most significantly represented genes we identified for QTL loci are primarily associated with the glycoside hydrolase family and ceramide biosynthetic process functions.

The list of genes associated with glycoside hydrolase function and ceramide biosynthetic process, together with the locus to which they belong, is given in **Supplementary Table S14** (**Supplementary File 2**). This table shows that 40% of genes related to glycoside hydrolase activity (9 out of 23) are located in the QTL region on chromosome 3D, at position 102 cM. This position was previously noted for its overlap with a large number of QTLs for the color trait (**Supplementary Table S10**, **Supplementary File 2**). This region is associated with the largest number of texture traits, nine, which indicates its high significance. According to **Figure 2**, some traits associated with roughness are among these QTLs: *Q.GLCMi-3D^4^* (GLCM inertia), *Q.GLCMmp-3D^4^* (GLCM max probability), *Q.GLCMc-3D^4^* (GLCM correlation), *Q.PC1-3D^4^* (first principal component). At the same time, two traits, GLCMi and GLCMc, are closely related to grain shell lightness traits (**Figure 1**; **Supplementary Table S5**, **Supplementary File 1**). Eight of the 23 glycoside hydrolase genes were localized in the QTL region on chromosome 4D, position 164.488. Interestingly, this region corresponds to two QTLs also associated with grain roughness/smoothness traits: *Q.GLCMс-4D^5^* (GLCM correlation) and *Q.GLCMi-4D^5^* (GLCM inertia), which are associated with grain shell lightness traits.

Most of the genes associated with the ceramide biosynthetic process are located on chromosome 5B, position 75 cM (4 out of 7). Two genes are located on chromosome 4D and one on chromosome 3D.

KEGG orthologous groups were identified for 531 of the 1,213 genes. Analysis of the KEGG annotation showed that a significant proportion of the genes (almost half) belong to the functional category “Genetic information processing.” The next categories are ‘Carbohydrate metabolism’, ‘Signaling and cellular processes’, ‘Environmental information processing’, and ‘Lipid metabolism’. Based on KO identifiers, 35 genes associated with seed development processes were identified. Their list is given in **Supplementary Table S15** (**Supplementary File 2**). They are represented in all loci we detected, except for chromosome 3B. Most (14 genes) are located in the 4D region of the chromosome, at position 164.488 cM. The genes associated with seed development include transcription factors (MADS-box, EREBP-like, MYB, HD-ZIP, AP2-like, NFYC, 19 genes in total), while the remaining genes encode various enzymes, kinases, translation regulators, and a number of others. Based on these data, it is difficult to identify any functional group specific to these genes.

## 4 Discussion

The texture of grains in an image is a complex feature that depends on many factors. The perception of texture depends on the position of the observer, lighting, and the characteristics of the object’s surface (Désage et al., 2015). The characteristics of an object’s surface are determined by its color, material structure, and relief. Digital representation of texture in images is a complex task, and none of the many descriptors used to evaluate it provide a complete representation (Bianconi et al., 2021). For example, the GLCM (Haralick et al., 1973) and GLRM (Galloway, 1975) descriptors used in this work evaluate the spatial variability of only the intensity of image illumination, but not color.

It can be assumed that the texture properties of grains are determined by the presence of pigments and their distribution in the shell, as well as by the structure of the external and internal tissues of the grain. Inter- and intraspecific differences in grains based on these characteristics are so pronounced that the use of texture characteristics allows for highly accurate classification of plant grains into species (Majumdar and Jayas, 2000c) or varieties (Ropelewska and Rutkowski, 2021).

This study performed a comprehensive analysis of grain texture characteristics in images of wheat samples from the ITMI population. As in many previous studies, significant diversity in grain texture was observed among the samples. However, the results obtained in this study allowed the identification of two main types of grain shell variability: roughness/smoothness and wrinkling along/across the grain axis (**Figure 2**). These characteristics are well known for grains. The wrinkling of pea seeds was observed by Mendel in his pioneering work on genetics (Williams et al., 2024). In the work of Jabeen (Jabeen et al., 2023), significant interspecific differences were found in the grains of the genus *Salvia* L. in terms of both smoothness (smooth/scabrous) and roughness. An analysis of a large number of morphological characteristics of grains in a corn population demonstrated significant differences, including texture traits (Wang et al., 2025).

Using grains from different samples and harvest years as examples, in the present study it was shown that genetic components and harvest year make a significant contribution to the diversity of all 16 texture characteristics of wheat grains. Thus, like most other wheat grain traits, such as size, shape, and color (Arif et al., 2021, Arif et al., 2022b), texture characteristics are influenced by both genetic and environmental factors.

The study shows that grain texture characteristics are significantly related to the duration of their storage in the genbank. Previously, we discovered a similar relationship for the color characteristics of grains from the same wheat population (Afonnikov et al., 2022). The dependence of color on the duration of grain storage may be partly related to gradual metabolic changes in the grain shell, leading to changes in pigment concentration (Shvachko and Khlestkina, 2020; Pirredda et al., 2023). Apparently, similar changes may occur in the microstructure of the grain shell. Interestingly, these changes are characterized by an increase in the roughness. This may be due to the degradation of certain structural components of the shell, occurring against the background of numerous biochemical and structural changes in the grains during aging (Nagel and Börner, 2010; Arif et al., 2022a). In particular, it is known that one of the characteristic changes in the internal structure of grains subjected to long-term storage is cell shrinkage (Nadarajan et al., 2023), which can potentially lead to changes in the surface structure of the grain. Another possible factor may be changes in the cell wall or the destruction of mucilage during storage (Sano et al., 2016), which may lead to exfoliation of the cell wall and an increase in its roughness. However, it is still challenging to make a definitive judgment about the mechanisms of the observed variability.

In our work, a reliable correlation between texture characteristics and grain germination was found for only one characteristic, CLCM correlation. It is weak, unlike the redness traits for the same population (Afonnikov et al., 2022), for which the correlation coefficient with germination reached absolute values ranging from 0.164 to 0.235, while the threshold values for randomization tests in most cases did not exceed 0.1 in absolute terms, as in the present work. Another trait, CLCM inertia, showed significant deviations from zero only in the bootstrap test. Thus, the relationship between texture and germination is weak. However, it is only evident for traits that correlate with grain lightness and roughness.

A significant correlation between grain coat texture traits and seed emergence rate was observed for corn (Wang et al., 2025). However, a recent analysis of peas showed that seed dormancy is genetically separable from seed coat thickness and roughness (Williams et al., 2024). These results are contradictory, which may be due to the influence of a whole complex of factors affecting grain germination: the structure and water permeability of the coat, pigment concentration, hormone activity, and many others (Han and Yang, 2015; Steinbrecher and Leubner-Metzger, 2017; Farooq et al., 2022). These factors may contribute differently to different plant species. Obviously, more detailed study is needed to answer the question of the relationship between germination and the texture of the seed coat of different species.

Our work has identified several QTLs for grain texture traits, both additive and epistatic. For grain traits obtained from image analysis, QTL and GWAS analyses are widely used to search for genes involved in their control in cereals and other plant species (Jamil et al., 2025). However, to our knowledge, such analysis has not previously been performed for cereal grain texture traits. The presence of significant QTLs confirms the genetic basis of texture traits. The results obtained in this study show that, as in the case of digital traits of grain color, size, and shape (Arif et al., 2022b; Afonnikova et al., 2024), for many QTLs, the location of loci in the genome coincides. This means that the same genes influence the formation of multiple grain characteristics simultaneously (both due to the statistical dependence of the traits themselves and due to the biological mechanisms that determine them). A comparison of the location of QTLs for sets of characteristics of size, shape, color, and texture showed that there is no overlap between the loci of texture and grain size/shape, but for several regions of the genome, the QTLs of color and texture overlap. The locus associated with the largest number of wheat grain color/texture traits (a total of 34 color traits and 10 texture traits) is located on chromosome 3D at positions 100–102 cM at a distance of ~1. 5 Mb from the TaMYB10 gene (Lang et al., 2024), which is involved in the regulation of grain color and control of pre-harvest sprouting. This is noteworthy because, in a recent analysis of pea grain traits related to shell structure (testa thickness and permeability), the loci of these quantitative traits were found to be closely associated with Mendel’s pigmentation locus A (Williams et al., 2024).

Modern methods of analyzing high-density marker genetic maps allow for the prioritization of genes from QTL regions in the wheat genome (Bargsten et al., 2014; Rezaei et al., 2021; Arif et al., 2022b; Afonnikova et al., 2024). For texture traits, two functional groups of genes localized in significant QTL regions were identified in the current study: those associated with glycoside hydrolase family 17 and ceramide biosynthetic process. Glycoside hydrolase family 17 comprises enzymes with several known activities (Henrissat and Davies, 2000; Minic and Jouanin, 2006; Minic, 2008): endo-1,3-*β*-glucanase (EC 3.2.1.6), endo-1,4-*β*-glucanase (EC 3.2.1.74). In plants, glycoside hydrolases family is involved in the degradation of cell wall polysaccharides (Minic and Jouanin, 2006). They are also involved in starch sucrose and raffinose metabolism, seed development in Arabidopsis (Minic, 2008). Proteins of this family were detected experimentally in the developing wheat endosperm (Suliman et al., 2013). In wheat, glycoside hydrolase family 17 (GH17) comprises 209 genes representing four groups of clades in phylogenetic tree (Penning, 2023). Genes of this family involved in wheat defense against *R. cerealis* and can inhibit activity of additional pathogenic fungi of rice, hot pepper and tobacco (Liu et al., 2009). Expression of these genes has inhibitory effect on fungi commonly associated with wheat kernel (Zhang et al., 2019). Plant β-1,3-glucanases also play a role in the degradation of callose in plasmodesmata which form channels physically interconnecting the cytoplasm and endoplasmic reticulum of adjacent cells (Perrot et al., 2022). Interestingly, reversible callose accumulation is known to be involved in regulating symplastic connectivity of plant cells and adjacent epidermal cells at different stages of plant development (Zavaliev et al., 2011).

Ceramides are the basic unit of all sphingolipids signaling molecules involved in many processes in plants (Lynch and Dunn, 2004; Pata et al., 2010; Mehta et al., 2021; Liu et al., 2021), such as plant disease or defense (Berkey et al., 2012), cell membrane architecture formation and membrane trafficking (Mamode Cassim et al., 2020). Ceramides abundant in seeds as was demonstrated in durum wheat (Cutignano et al., 2021) and soybean (Gao et al., 2025). Their abundance changes with seed development (Wang et al., 2006). As in the case of the glycoside hydrolase family, the function of these metabolites is also related to the cell wall. Interestingly, in Arabidopsis sphingolipids together with sterols are highly enriched in plasmodesmata (Grison et al., 2015). It was demonstrated that the modulation of the overall sterol composition of young dividing cells reversibly impaired the plasmodesmata localization of the glycosylphosphatidylinositol anchored proteins, including β-1,3-glucanases, resulting in altered callose-mediated permeability (Grison et al., 2015). This allows us to hypothesize that the structure/functions of cell walls and cell-to-cell connectivity are somehow related to the structure of the grain surface and, consequently, to its texture characteristics.

Current and our previous works (Afonnikov et al., 2022; Arif et al., 2022b) demonstrated usefulness and efficiency of digital image processing in analysis of morphological, physiological wheat grain characteristics and their genetic control. Results of these works are summarized in (**Table 3**).

**Table 3:** Genetic control and relationships with physiological characteristics of the wheat grain size, shape, color and texture traits obtained from digital images.

Type of traits	Size/shape	Color	Texture
Number of traits^1^	7	48	16
Number of QTLs^2^	42	170	36
Number of chromosomes with QTLs^2^	15	16	8
Number of genomic regions^2,3^	19	21	9
Number of traits correlated with storage duration^1^	1	44	14
Number of traits correlated with germination rate^1^	0	7	1

^1^this work and Afonnikov et al. (2022);

^2^this work and Arif et al. (2022b);

^3^LOD>3, left and right markers mapped to the genome.

They demonstrated that genetic control of these traits are complex: more than two QTLs per trait were identified; they are located in different wheat chromosomes. Size and shape properties do not change with grain storage duration unlike color traits. Size and shape properties have no relationship with seed germination unlike color traits describing redness (Afonnikov et al., 2022). Interestingly, texture traits are highly related to grain storage duration but demonstrate absence of the relationship to germination rate like size and shape traits.

The lack of clarity in the grain texture features in the images makes it hard to find connections between them and the grain properties that can be described at the molecular, metabolic, or structural level. Perhaps such a connection can be established by searching for correlations between texture and other grain properties (color, mechanical, biochemical, etc.). For example, in a study (Wang et al., 2025), it was shown that grain texture descriptors displayed significant correlations with light transmissivity parameters determined using a hyperspectral sensor, several of them negative (texture smoothness, texture repetition, and pixel correlation) and one positive (texture roughness). Significant relationships were found between texture and visible color descriptors of seed coat (similar to our results). No significant relationship was found for texture characteristics and seed surface roughness estimated using atomic force microscopy.

From the other hand, more detailed description of texture can be obtained by using additional characteristics. For example, there are methods that take into account textural features for different color spaces, which can describe texture and its dependence on object color in greater detail (Ropelewska and Rutkowski, 2021; Ropelewska et al., 2022). They may reveal more subtle associations between texture and color characteristics and identify more QTLs for further analysis. A larger number of traits will allow describing more characteristics of the shell. This will allow to more reliably search for associations with genetic variations in the complex of grain texture traits.

## 5 Conclusion

Here, a comprehensive analysis of the texture of soft wheat grains in digital images for plants from the ITMI population is presented. It allowed to characterize the variability of accessions in terms of texture and demonstrated two main directions of texture variability related to grain roughness/smoothness and wrinkling. It was shown that both genotype and the factor of storage duration in the genbank contribute significantly to the formation of grain texture characteristics. The relationship between texture traits and grain germination was found only for one characteristic, GLCM correlation, and was found to be weak. The QTLs we identified, both additive and epistatic, which demonstrate that texture traits are controlled by several loci located on eight chromosomes. The location of some of these QTLs in the genome overlaps with loci involved in grain color control. Prioritization of genes in the identified loci and their functional analysis allowed us to hypothesize a possible link between texture traits and cell wall properties. Overall, our analysis showed the complex nature of wheat grain characteristics such as surface texture. Further study will shed light on the genetic mechanisms that underlie the formation of plant grain texture traits.

## Data Availability

The original contributions presented in the study are included in the article/**Supplementary Material**. Further inquiries can be directed to the corresponding author.
