# Supplementary material for: Analysis of coat texture characteristics of bread wheat grains obtained from digital images
Source: Front Plant Sci. 2025 Sep 30;16:1659548. doi: 10.3389/fpls.2025.1659548 (PMC12518363; doi:10.3389/fpls.2025.1659548)
Supplement: Supplementary file 1 [file DataSheet1.pdf]

Supplementary file 1

Table S1. List of seed traits obtained from the images and used in analysis

Trait	Description	Trait
**Size traits**		
1	sL	Seed length
2	sW	Seed width
3	sA	Seed area
**Shape traits**		
4	sCi	Seed shape circularity
5	sRo	Seed shape roundness
6	sRu	Seed shape rugosity
7	sSo	Seed shape solidity
**Color traits**		
8	RGB_mR	Mean R component for seed pixels
9	RGB_mG	Mean G component for seed pixels
10	RGB_mB	Mean B component for seed pixels
11	HSV_mH	Mean H component for seed pixels
12	HSV_mS	Mean S component for seed pixels
13	HSV_mV	Mean V component for seed pixels
14	Lab_mL	Mean L* component for seed pixels
15	Lab_ma	Mean a* component for seed pixels
16	Lab_mb	Mean b* component for seed pixels
17	YCrCb_mY	Mean Y component for seed pixels
18	YCrCb_mCr	Mean Cr component for seed pixels
19	YCrCb_mCb	Mean Cb component for seed pixels
**Texture traits**		
20	GLCMcp	GLCM cluster prominance
21	GLCMcs	GLCM cluster shade
22	GLCMc	GLCM correlation
23	GLCMe	GLCM entropy
24	GLCMh	GLCM homogeneity
25	GLCMi	GLCM inertia
26	GLCMmp	GLCM max probability
27	GLCMm	GLCM mean
28	GLCMu	GLCM uniformity
29	GLCMv	GLCM variance
30	GLRMe	GLRM entropy
31	GLRMglnu	GLRM gray level non uniformity
32	GLRMlr	GLRM long run
33	GLRMrlnu	GLRM run length non uniformity
34	GLRMrr	GLRM run ratio
35	GLRMsr	GLRM short run

Table S2. Descriptions and equations for calculation of second order gray level matrices for texture traits evaluation.

Eq.	Feature (abbreviation)	Equation	Reference
(1)	Gray level co-occurrence matrix (GLCM)	$P_d(i,j) = \sum_{x=1}^{n}\sum_{y=1}^{m}\sum_{\Delta x,\Delta y}\begin{cases}1, \text{if } I(x,y) = i \text{ and } I(x+\Delta x, y+\Delta y) = j \\ \text{and } \delta(\Delta x, \Delta y) = d; \\ \text{otherwise}, 0;\end{cases}$<br>where<br>$x$, y are the coordinates of pixels in the image; $0 < x < n$, $0 < y < m$; $i, j = 0, \ldots, k-1$; k is the number of levels of image luminance quantization; $\delta(\Delta x, \Delta y)$, the distance between adjacent pixels (x, y) and $(x+\Delta x, y+\Delta y)$; $I(x, y)$, pixel intensity in gray scale; Δx, the shift along the X axis; and Δy, the shift along the Y axis.<br>Normalized matrix<br>$p(i,j) = \frac{\sum_x^n \sum_y^m P_{x,y}(i,j)}{C}$,<br>where<br>$C = 2n(m-1) + 2m(n-1) + 4(n-1)(m-1)$	Haralick et al., 1979
(2)	Gray level run length matrix (GLRM)	$q(i,j) = \sum_\theta Q_\theta(i,j)$,<br>where<br>$Q_\theta(i,j)$ is the number of runs of length j of the pixels with the gray level i in the direction θ from pixel (i,j)	Galloway, 1974

Table S3. Characteristics computed from the normalized GLCM matrix, where $p(i,j)$ is its element in the ith row and jth column according to Majumdar and Jayas (1999).

Name	Description	Equation
GLCM mean	The GLCM mean represents the average intensity value of pixel pairs contributing to the co-occurrence matrix. It quantifies the central tendency of gray-level distributions in neighboring pixel relationships.	$\sum_{i=1}^{N_g}\sum_{j=1}^{N_g} i p_{i,j}$
GLCM variance	Variance measures the dispersion of neighboring intensity pairs around the mean value in the GLCM. Higher values indicate greater heterogeneity in local intensity relationships.	$\sum_{i=1}^{N_g}\sum_{j=1}^{N_g} (i-\mu)^2 p_{i,j}$
GLCM uniformity	This metric, also called energy, reflects the homogeneity of texture patterns. Elevated values occur when specific gray-level pairs dominate the GLCM, signaling repetitive structural arrangements.	$\sum_{i=1}^{N_g}\sum_{j=1}^{N_g} {p_{i,j}}^2$
GLCM entropy	Entropy quantifies the randomness or complexity of gray-level pair distributions. High entropy corresponds to irregular textures with diverse intensity relationships, while low entropy indicates uniform patterns.	$\sum_{i=1}^{N_g}\sum_{j=1}^{N_g} p_{i,j}\left(p_{i,j}\right)$
GLCM maximum probability	This identifies the most frequent gray-level pair in the GLCM. Dominant pixel combinations produce peaks in the matrix, reflecting prevalent local intensity relationships	$p_{i,j}$
GLCM correlation	Correlation assesses linear dependencies between neighboring pixel intensities. It highlights structural directionality and periodic patterns in textures.	$\sum_{i=1}^{N_g}\sum_{j=1}^{N_g} p_{i,j}\left[\frac{(i-\mu)(j-\mu)}{\sigma^2}\right]$
GLCM homogeneity	Homogeneity emphasizes pixel pairs with similar intensities, assigning higher weights to diagonal GLCM elements. It increases with local uniformity and decreases with contrast.	$\sum_{i=1}^{N_g}\sum_{j=1}^{N_g} \frac{p_{i,j}}{1+(i-j)^2}$
GLCM inertia	Contrast measures intensity variations between neighboring pixels, emphasizing off-diagonal GLCM elements. It grows with abrupt transitions and edge density in textures	$\sum_{i=1}^{N_g}\sum_{j=1}^{N_g} p_{i,j}(i-j)^2$
GLCM cluster shade	Cluster shade evaluates the asymmetry of GLCM element distribution around the mean. Positive values indicate right-skewed distributions, while negative values suggest left-skewed patterns.	$\sum_{i=1}^{N_g}\sum_{j=1}^{N_g} (i+j-2\mu)^3 p_{i,j}$
GLCM cluster prominence	This metric quantifies the peakedness of GLCM element distribution relative to the mean. Higher values imply flatter distributions with outliers, whereas lower values denote concentration near the mean.	$\sum_{i=1}^{N_g}\sum_{j=1}^{N_g} (i+j-2\mu)^4 p_{i,j}$

Table S4. Characteristics computed from the GLRM matrix, where $q(i, j)$ is its element in the ith row and jth column according to Majumdar and Jayas (1999).

Name	Description	Equation
GLRM short run	Short-run emphasis quantifies the prevalence of brief intensity sequences, correlating with fine-grained textures. It decreases as longer runs dominate the image.	$\sum_{i=1}^{N_g}\sum_{j=1}^{N_r}(q(i,j)/j^2)/R$
GLRM long run	Long-run emphasis highlights coarse textures dominated by extended sequences of identical intensities. It increases with the prevalence of lengthy homogeneous regions.	$\sum_{i=1}^{N_g}\sum_{j=1}^{N_r}(j^2 q(i,j))/R$
GLRM gray level non-uniformity	This evaluates the variability of run lengths within individual gray levels. Lower values indicate consistency in run distributions per intensity, while higher values signal irregularity.	$\sum_{i=1}^{N_g}\left(\sum_{j=1}^{N_r} q(i,j)\right)/R^{\square}$
GLRM run length non-uniformity	This assesses the diversity of run lengths across all gray levels. Uniform textures yield low values, whereas varied run lengths produce higher measurements.	$\sum_{j=1}^{N_r}\left(\sum_{i=1}^{N_g} q(i,j)\right)/R^{\square}$
GLRM run ratio	Run ratio characterizes texture coarseness by comparing total runs to pixel count. Higher ratios indicate frequent short runs, typical of fine textures, while lower ratios suggest longer runs.	$R/\sum_{i=1}^{N_g}\sum_{j=1}^{N_r} jq(i,j)$
GLRM entropy	Entropy in GLRM measures randomness in run-length distributions across gray levels. It increases with heterogeneous run-length patterns and decreases with uniform textural repetitions.	$\sum_{i=1}^{N_g}\sum_{j=1}^{N_r} q(i,j)log(q(i,j))/R$

REFERENCES

1. Haralick R. M., Shanmugam K., Dinstein I. H. Textural features for image classification. IEEE Transactions on systems, man, and cybernetics. 1973; 6: 610-621.
2. Galloway M. M. Texture analysis using grey level run lengths. STIN. 1974; 75: 18555.
3. Majumdar S., Jayas D. S. Classification of bulk samples of cereal grains using machine vision. Journal of Agricultural Engineering Research. 1999; 73(1): 35-47. DOI 10.1006/jaer.1998.0388

Table S5. Pearson correlation coefficients (lower left corner) for seed traits in the ITMI population of the 2014 harvest. The most significant positive values are shown in red, negative values are shown in blue. The upper right triangle contains *p*-values, the non-significant ones are highlighted with a pink background.

	RGB_mR	RGB_mG	RGB_mB	HSV_mH	HSV_mS	HSV_mV	Lab_mL	Lab_ma	Lab_mb	YCrCb_mY	YCrCb_mCr	YCrCb_mCb	sL	sW	sA	sCi
RGB_mR		0	0	0	0	0	0	0	3.18E-07	0	0	0.00	0.00	0.34	0.01	0.03
RGB_mG	0.94		0.00	0.00	0.00	0.00	0.00	0.00	0	0	0	0.89	0.00	0.07	0.00	0.04
RGB_mB	0.78	0.92		0.00	0.00	0.00	0.00	0.00	0	0	0	0.00	0.01	0.01	0.00	0.23
HSV_mH	0.60	0.70	0.48		0.00	0.00	0.00	0.00	0	0	0	0.00	0.02	0.78	0.07	0.00
HSV_mS	-0.50	-0.73	-0.93	-0.32		0.00	0.00	0.00	0	0	0	0.00	0.05	0.01	0.00	0.03
HSV_mV	1.00	0.94	0.78	0.60	-0.49		0.00	0.00	1.68E-07	0	0	0.00	0.00	0.32	0.01	0.03
Lab_mL	0.96	1.00	0.90	0.68	-0.69	0.96		0.00	1.49E-06	0	0	0.01	0.00	0.12	0.00	0.04
Lab_ma	-0.54	-0.79	-0.82	-0.77	0.83	-0.54	-0.74		0	0	0	0.00	0.03	0.01	0.00	0.04
Lab_mb	0.11	-0.16	-0.52	0.23	0.78	0.11	-0.10	0.43		5.08E-10	0	0.00	0.79	0.00	0.06	0.00
YCrCb_mY	0.96	1.00	0.92	0.66	-0.71	0.96	1.00	-0.75	-0.13		0	0.21	0.00	0.09	0.00	0.08
YCrCb_mCr	-0.21	-0.53	-0.73	-0.42	0.89	-0.20	-0.46	0.89	0.78	-0.48		0.00	0.22	0.00	0.00	0.45
YCrCb_mCb	-0.26	0.00	0.38	-0.34	-0.67	-0.26	-0.06	-0.32	-0.99	-0.03	-0.70		0.44	0.01	0.17	0.00
Sl	-0.07	-0.07	-0.06	-0.05	0.04	-0.07	-0.07	0.05	-0.01	-0.07	0.03	0.02		0.00	0.00	0.00
sW	-0.02	-0.04	-0.06	-0.01	0.06	-0.02	-0.03	0.06	0.06	-0.04	0.06	-0.06	0.20		0.00	0.03
sA	-0.06	-0.07	-0.07	-0.04	0.07	-0.06	-0.07	0.07	0.04	-0.07	0.06	-0.03	0.79	0.76		0.00
sCi	-0.05	-0.04	0.03	-0.19	-0.05	-0.05	-0.04	0.04	-0.15	-0.04	-0.02	0.15	-0.21	-0.05	-0.15	
sRo	0.04	0.02	0.00	0.03	0.01	0.04	0.03	0.01	0.06	0.02	0.03	-0.06	-0.65	0.60	-0.05	0.16
sRu	0.10	0.10	0.04	0.19	-0.01	0.10	0.10	-0.07	0.11	0.10	-0.01	-0.13	-0.07	0.27	0.11	-0.85
sSo	-0.05	-0.05	0.01	-0.19	-0.03	-0.05	-0.05	0.06	-0.13	-0.05	0.00	0.14	-0.01	-0.14	-0.06	0.90
GLCMm	0.42	0.44	0.40	0.30	-0.31	0.42	0.44	-0.34	-0.05	0.44	-0.21	-0.02	-0.18	0.08	-0.06	0.19
GLCMv	0.30	0.32	0.31	0.20	-0.25	0.30	0.32	-0.25	-0.08	0.32	-0.18	0.03	0.13	-0.12	0.01	-0.22
GLCMu	0.54	0.54	0.42	0.46	-0.26	0.54	0.54	-0.40	0.11	0.54	-0.17	-0.20	-0.17	0.12	-0.03	0.07
GLCMe	-0.28	-0.27	-0.22	-0.21	0.15	-0.28	-0.28	0.19	-0.04	-0.28	0.08	0.08	-0.11	0.09	-0.01	0.23
GLCMmp	0.50	0.51	0.41	0.46	-0.28	0.50	0.52	-0.40	0.08	0.51	-0.20	-0.16	-0.16	0.10	-0.04	-0.08
GLCMc	0.45	0.45	0.38	0.33	-0.26	0.45	0.45	-0.30	0.03	0.45	-0.15	-0.10	0.04	0.04	0.05	-0.21
GLCMh	0.47	0.46	0.34	0.43	-0.20	0.47	0.47	-0.32	0.14	0.46	-0.12	-0.22	-0.19	0.21	0.01	-0.03
GLCMi	-0.54	-0.52	-0.41	-0.42	0.26	-0.54	-0.53	0.34	-0.11	-0.52	0.14	0.19	0.01	-0.10	-0.06	0.12
GLCMcs	0.24	0.26	0.26	0.15	-0.22	0.24	0.26	-0.20	-0.09	0.26	-0.16	0.05	0.16	-0.14	0.01	-0.22
GLCMcp	0.20	0.22	0.23	0.12	-0.19	0.20	0.21	-0.17	-0.09	0.22	-0.14	0.06	0.17	-0.16	0.01	-0.22
GLRMsr	-0.34	-0.33	-0.25	-0.28	0.16	-0.34	-0.33	0.22	-0.08	-0.33	0.10	0.13	0.14	-0.20	-0.03	0.13
GLRMlr	0.37	0.37	0.29	0.31	-0.18	0.37	0.37	-0.25	0.08	0.37	-0.11	-0.14	-0.12	0.21	0.05	-0.11
GLRMglnu	0.19	0.18	0.14	0.14	-0.09	0.19	0.19	-0.13	0.03	0.18	-0.05	-0.06	0.08	0.08	0.11	0.16
GLRMrlnu	-0.04	-0.06	-0.05	-0.06	0.06	-0.04	-0.05	0.06	0.02	-0.05	0.06	-0.01	0.13	0.05	0.12	0.20
GLRMrr	-0.38	-0.37	-0.28	-0.31	0.18	-0.38	-0.37	0.25	-0.08	-0.37	0.11	0.14	0.12	-0.21	-0.05	0.10
GLRMe	0.13	0.12	0.09	0.09	-0.05	0.13	0.13	-0.08	0.03	0.12	-0.02	-0.05	0.10	0.05	0.10	0.18

	sRo	sRu	sSo	GLCMm	GLCMv	GLCMu	GLCMe	GLCMmp	GLCMc	GLCMh	GLCMi	GLCMcs	GLCMcp	GLRMsr	GLRMlr	GLRMglnu	GLRMrlnu	GLRMrr	GLRMe
RGB_mR	0.07	7.5E-07	0.02	0	0	0	0	0	0	0	0	0	0	0	0	0	0.07	0	6.78E-10
RGB_mG	0.28	0.00	0.01	0	0	0	0	0	0	0	0	0	0	0	0	0	0.01	0	8.1E-09
RGB_mB	0.94	0.06	0.63	0	0	0	0	0	0	0	0	0	0	0	0	0	0.01	0	1.3E-05
HSV_mH	0.12	0.00	0.00	0	0	0	0	0	0	0	0	0	1.53E-08	0	0	0	0.00	0	1.12E-05
HSV_mS	0.49	0.55	0.20	0	0	0	0	0	0	0	0	0	0	0	0	3.44E-05	0.01	0	0.02
HSV_mV	0.07	0.00	0.02	0	0	0	0	0	0	0	0	0	0	0	0	0	0.06	0	7.12E-10
Lab_mL	0.19	0.00	0.01	0	0	0	0	0	0	0	0	0	0	0	0	0	0.01	0	2.49E-09
Lab_ma	0.52	0.00	0.01	0	0	0	0	0	0	0	0	0	0	0	0	3.22E-10	0.00	0	7.59E-05
Lab_mb	0.01	0.00	0.00	0.01	0.00	0.00	0.10	0.00	0.16	0.00	0.00	0.00	0.00	0.00	0.00	0.16	0.34	6.28E-05	0.14
YCrCb_mY	0.24	0.00	0.03	0	0	0	0	0	0	0	0	0	0	0	0	0	0.01	0	4.48E-09
YCrCb_mCr	0.11	0.67	0.93	0	0	0	7.46E-05	0	0	1.04E-08	1.74E-10	0	0	5.81E-06	1.06E-07	0.010362	0.01	4.41E-07	0.27
YCrCb_mCb	0.00	0.00	0.00	0.47	0.15	0.00	0.00	0.00	0.00	0.00	0.00	0.03	0.01	0.00	0.00	0.00	0.51	0.00	0.01
Sl	0.00	0.00	0.67	0.00	0.00	0.00	0.00	0.00	0.09	0.00	0.65	0.00	0.00	0.00	0.00	0.00	0.00	0.00	7.32E-06
sW	0.00	0.00	0.00	0.00	0.00	0.00	0.00	0.00	0.07	0.00	0.00	0.00	0.00	0.00	0.00	0.00	0.02	0.00	0.02
sA	0.02	0.00	0.00	0.01	0.74	0.12	0.49	0.06	0.03	0.69	0.00	0.68	0.69	0.16	0.02	0.00	0.00	0.02	2.75E-06
sCi	0.00	0.00	0.00	0.00	0.00	0.00	0.00	0.00	0.00	0.15	0.00	0.00	0.00	0.00	0.00	0.00	0.00	0.00	0
sRo		0.00	0.00	0.00	0.00	0.00	0.00	0.00	0.98	0.00	0.00	0.00	0.00	0.00	0.00	0.85	0.00	0.00	0.12
sRu	0.25		0.00	0.96	0.00	0.01	0.00	0.00	0.00	0.00	0.00	0.00	0.03	0.00	0.00	0.00	0.00	0.00	0
sSo	-0.07	-0.84		0.00	0.00	0.40	0.00	0.00	0.00	0.00	0.00	0.00	0.00	0.00	0.00	0.00	0.00	0.00	0
GLCMm	0.21	0.00	0.14		0.00	0.00	0.00	0.00	0.00	0.00	0.00	0.00	0.00	0.00	0.00	0.00	0.08	0.00	0
GLCMv	-0.20	0.08	-0.18	-0.70		0.00	0.00	0.00	0.00	0.00	0.00	0.00	0.00	0.00	0.00	0.00	0.00	0.00	0.00
GLCMu	0.24	0.06	0.02	0.68	-0.33		0.00	0.00	0.56	0.00	0.00	0.00	0.00	0.00	0.00	0.00	0.65	0.00	0
GLCMe	0.16	-0.08	0.19	0.71	-0.94	0.17		0.05	0.00	0.00	0.00	0.00	0.00	0.00	0.02	0.00	0.00	0.00	0.25
GLCMmp	0.21	0.17	-0.13	0.44	-0.08	0.86	-0.04		0.00	0.00	0.00	0.00	0.00	0.00	0.00	0.00	0.02	0.00	5.33E-06
GLCMc	0.00	0.14	-0.20	-0.44	0.80	0.01	-0.85	0.20		0.00	0.00	0.00	0.00	0.00	0.00	0.01	0.00	0.00	0.03
GLCMh	0.31	0.19	-0.08	0.68	-0.35	0.84	0.26	0.76	0.07		0.00	0.00	0.00	0.00	0.00	0.00	0.01	0.00	0.00
GLCMi	-0.09	-0.11	0.12	0.08	-0.48	-0.37	0.59	-0.43	-0.83	-0.46		0.00	0.00	0.00	0.00	0.00	0.00	0.00	0.06
GLCMcs	-0.24	0.06	-0.17	-0.73	0.99	-0.38	-0.92	-0.12	0.77	-0.38	-0.46		0.00	0.00	0.00	0.00	0.00	0.00	0.00
GLCMcp	-0.27	0.05	-0.16	-0.74	0.97	-0.41	-0.90	-0.16	0.72	-0.40	-0.43	0.99		0.00	0.00	0.00	0.00	0.00	3.34E-05
GLRMsr	-0.27	-0.24	0.18	-0.41	0.16	-0.59	-0.09	-0.59	-0.21	-0.80	0.49	0.17	0.19		0.00	0.01	0.00	0.00	0
GLRMlr	0.25	0.22	-0.15	0.40	-0.13	0.63	0.05	0.64	0.24	0.82	-0.52	-0.15	-0.17	-0.96		0.00	0.00	0.00	0.48
GLRMglnu	0.00	-0.14	0.21	0.24	-0.13	0.31	0.07	0.21	-0.06	0.19	-0.08	-0.14	-0.15	0.06	0.11		0.00	0.00	0
GLRMrlnu	-0.06	-0.21	0.26	0.04	-0.08	0.01	0.08	-0.05	-0.12	-0.06	0.08	-0.08	-0.07	0.27	-0.11	0.90		0.00	0
GLRMrr	-0.26	-0.21	0.14	-0.42	0.15	-0.64	-0.07	-0.63	-0.22	-0.83	0.52	0.17	0.19	0.98	-1.00	-0.12	0.11		0.70
GLRMe	-0.03	-0.19	0.23	0.14	-0.07	0.19	0.02	0.10	-0.05	0.06	-0.04	-0.08	-0.09	0.20	-0.01	0.94	0.92	0.01	

Table S6. The results of the analysis of variance to assess the influence of genotype and harvest year factors on the average values of grain texture characteristics. The number of degrees of freedom *df*=43 for the genotype, *df* = 3 for the harvest year.

Texture characteristics	Genotype		Harvest year	
	F	*p*	*F*	*p*
GLCM_clProm	5.08	$2.45 \cdot 10^{-24}$	38.84	$1.12 \cdot 10^{-24}$
GLCM_clShade	5.19	$3.98 \cdot 10^{-25}$	20.79	$2.39 \cdot 10^{-13}$
GLCM_corr	12.88	$1.95 \cdot 10^{-82}$	150.07	$2.02 \cdot 10^{-91}$
GLCM_entropy	7.19	$8.75 \cdot 10^{-40}$	12.03	$7.78 \cdot 10^{-08}$
GLCM_homogeneity	29.31	$4.42 \cdot 10^{-198}$	165.89	$1.82 \cdot 10^{-100}$
GLCM_inertia	20.2	$1.12 \cdot 10^{-135}$	329.12	$5.69 \cdot 10^{-188}$
GLCM_maxProby	27.63	$6.90 \cdot 10^{-187}$	55.68	$3.84 \cdot 10^{-35}$
GLCM_mean	18.65	$1.28 \cdot 10^{-124}$	26.66	$4.86 \cdot 10^{-17}$
GLCM_uniformity	28.05	$1.11 \cdot 10^{-189}$	70.13	$5.05 \cdot 10^{-44}$
GLCM_variance	6.39	$7.32 \cdot 10^{-34}$	7.93	$2.86 \cdot 10^{-05}$
GLRM_entropy	1.89	0.00043	27.46	$1.53 \cdot 10^{-17}$
GLRM_gLNU	1.88	0.00047	32.29	$1.41 \cdot 10^{-20}$
GLRM_longRun	30.4	$3.04 \cdot 10^{-205}$	113.83	$2.72 \cdot 10^{-70}$
GLRM_rLNU	2.07	$5.77 \cdot 10^{-05}$	20.54	$3.42 \cdot 10^{-13}$
GLRM_runRatio	29.24	$1.32 \cdot 10^{-197}$	119.19	$1.87 \cdot 10^{-73}$
GLRM_shortRun	26.93	$3.14 \cdot 10^{-182}$	102.54	$1.38 \cdot 10^{-63}$

Table S7. Estimation of Pearson correlation coefficients (*r*) between grain texture characteristics and germination rate. The four right-hand columns show the minimum and maximum threshold values of the correlation coefficients, obtained on the basis of permutation and bootstrap tests. For correlation coefficients, the values showing significant deviations from 0 in comparison with randomization tests are highlighted in bold.

Trait	*r*	Permutation		Bootstrap	
		Min	Max	Min	Max
GLCMm	0.015	-0.101	0.107	-0.113	0.095
GLCMv	0.041	-0.095	0.088	-0.099	0.100
GLCMu	0.028	-0.082	0.089	-0.087	0.110
GLCMe	-0.037	-0.1	0.101	-0.093	0.093
GLCMmp	0.019	-0.116	0.086	-0.089	0.091
GLCMc	**0.098**	-0.101	**0.087**	-0.093	**0.097**
GLCMh	0.063	-0.088	0.092	-0.100	0.095
GLCMi	**-0.102**	-0.104	0.078	**-0.095**	0.108
GLCMcs	0.029	-0.089	0.09	-0.099	0.090
GLCMcp	0.016	-0.088	0.089	-0.103	0.105
GLRMsr	-0.051	-0.088	0.093	-0.087	0.087
GLRMlr	0.062	-0.098	0.105	-0.100	0.088
GLRMglnu	0.046	-0.106	0.097	-0.092	0.089
GLRMrlnu	0.052	-0.095	0.082	-0.095	0.093
GLRMrr	-0.061	-0.089	0.089	-0.103	0.101
GLRMe	0.048	-0.107	0.091	-0.086	0.089

B) ITMI_11

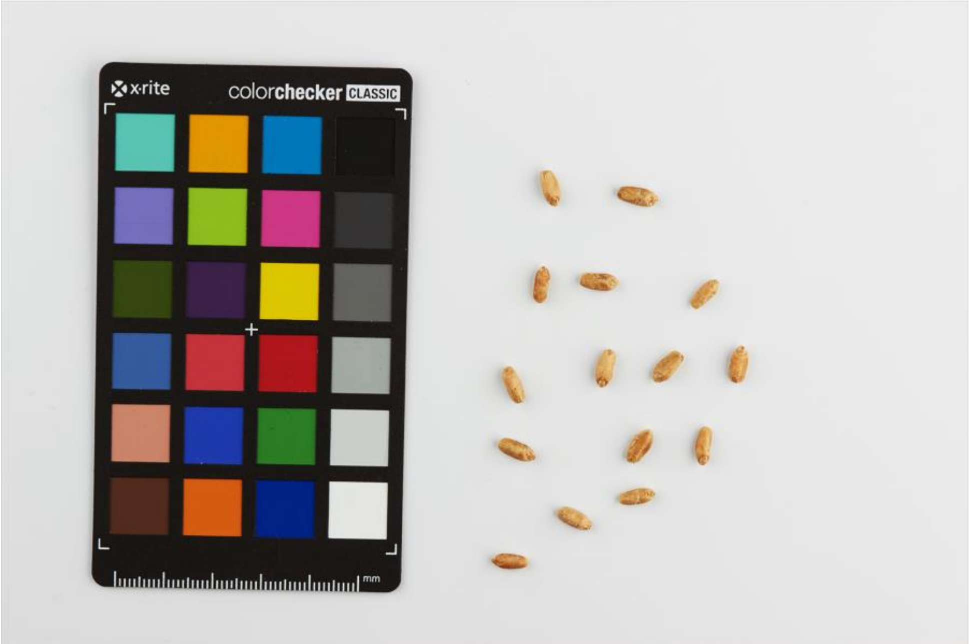

A) ITMI_62

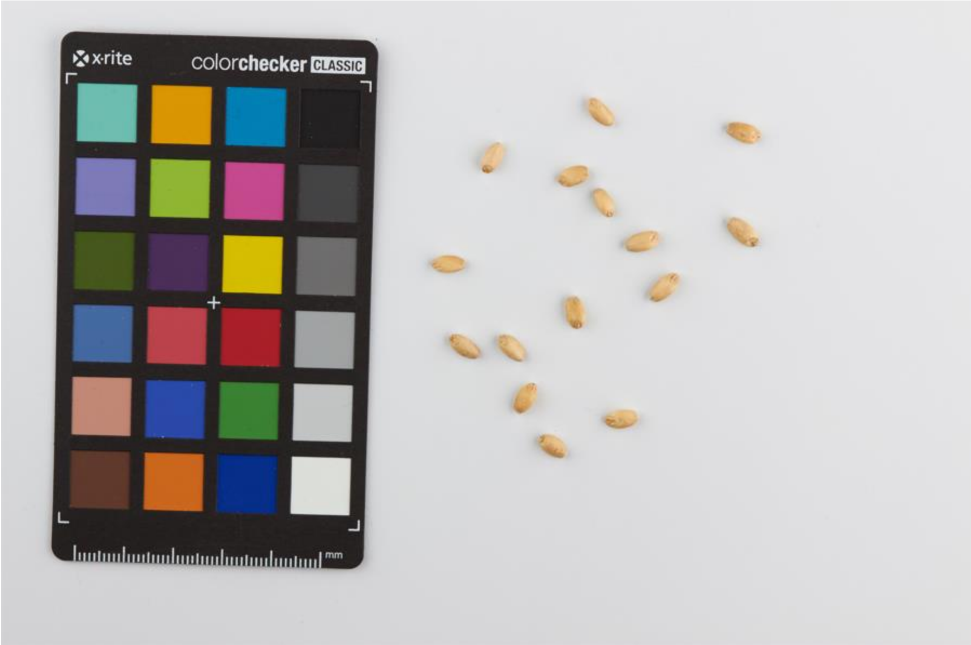

Figure S1. Examples of grain images for ITMI_11 (A) and ITMI_62 (B) RILs.

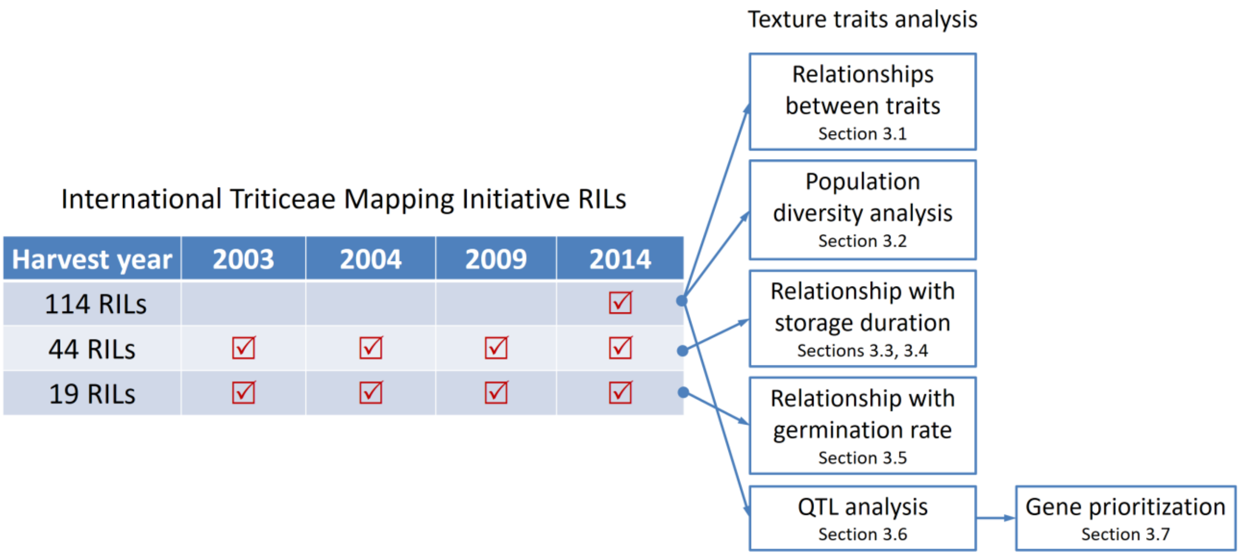

Figure S2. Analysis of grain coat texture characteristics based on International Triticeae Mapping Initiative line accessions harvested in different years.

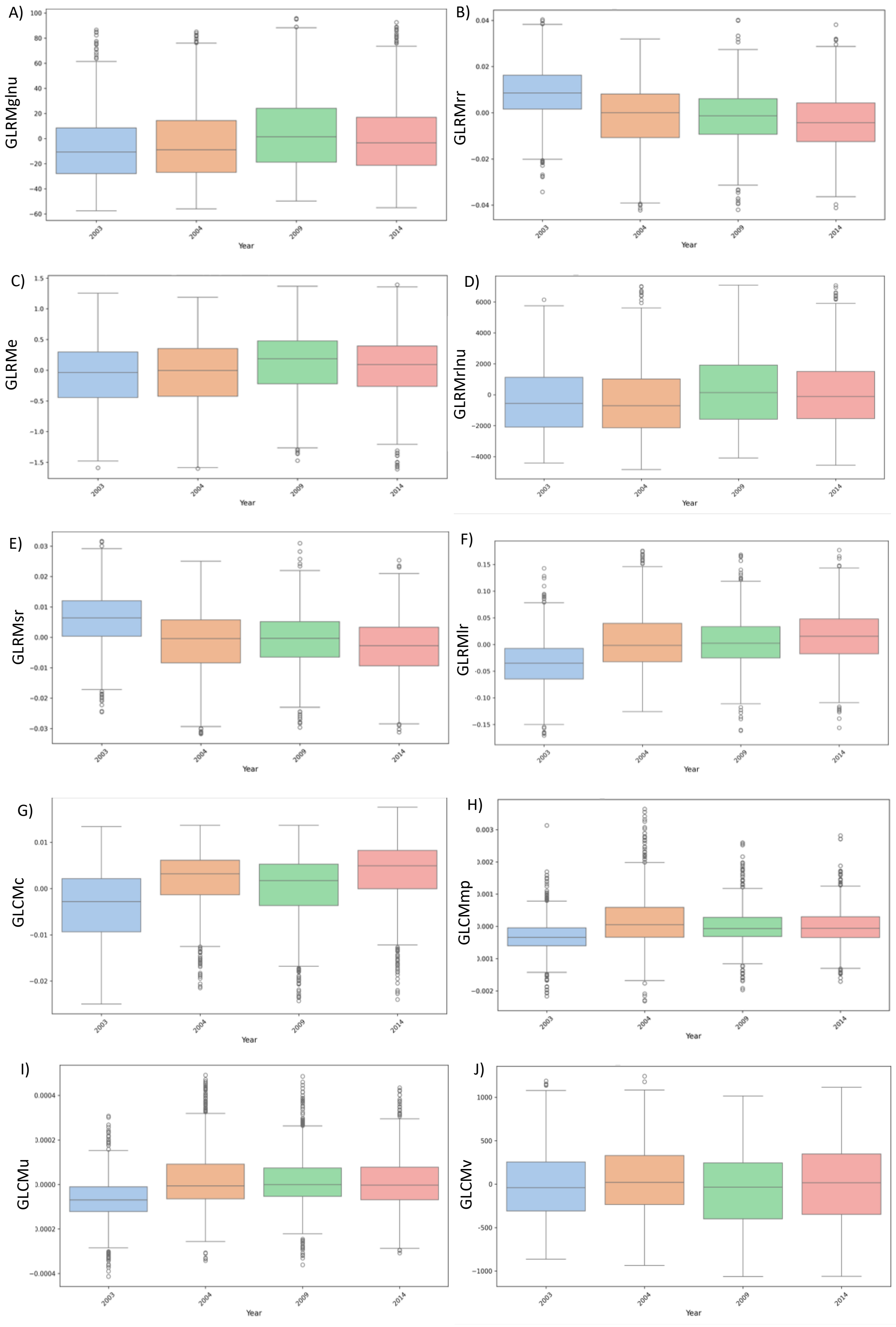

A)
GLRMglnu
B)
GLRMrr
C)
GLRMe
D)
GLRMrlnu
E)
GLRMsr
F)
GLRMlr
G)
GLCMc
H)
GLCMmp
I)
GLCMu
J)
GLCMv
Year
2003
2004
2009
2014

K)

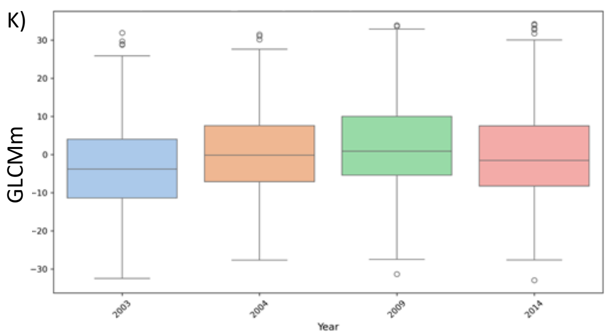

L)

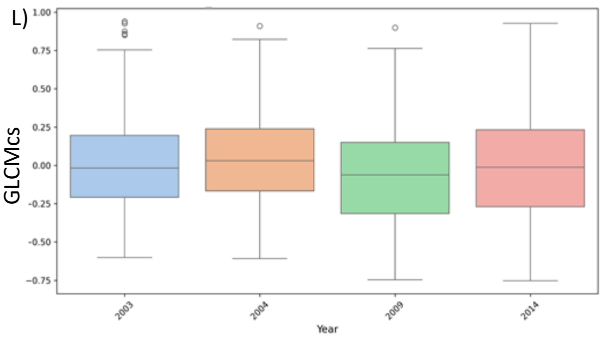

Figure S3. Bar plots of the dependence of the values of twelve textural features of wheat grains on the year of harvest. (A) GLRMglnu (GLRM gray level non uniformity); (B) GLRMrr (GLRM run ratio); (C) GLRMe (GLRM entropy); (D) GLRMrlnu (GLRM run length non uniformity); (E) GLRMsr (GLRM short run); (F) GLRMlr (GLRM long run); (G) GLCMc (GLCM correlation); (H) GLCMmp (GLCM max probability); (I) GLCMu (GLCM uniformity); (J) GLCMv (GLCM variance); (K) GLCMm (GLCM mean); (L) GLCMcs (GLCM cluster shade). The horizontal axis shows the storage years (2003, 2004, 2009, 2014), on the vertical axis are the values of the characteristics.
